# Sociodemographic predictors of PFAS exposure among a combined sample of U.S. pregnant women: an Environmental influences on Child Health Outcomes (ECHO) public-use dataset analysis

**DOI:** 10.1038/s41370-025-00833-8

**Published:** 2025-12-15

**Authors:** Jessie A. Gleason, Kristen Lyall, Jerald A. Fagliano, Lucy F. Robinson, Gloria B. Post, Anneclaire J. De Roos

**Affiliations:** 1 Department of Environmental and Occupational Health, Dornsife School of Public Health, Drexel University, Philadelphia, US; 2 Department of Epidemiology and Biostatistics, Dornsife School of Public Health, Drexel University, Philadelphia, US

## Abstract

**Background:**

Per- and polyfluoroalkyl substances (PFAS) are chemicals widely used in manufacturing since the 1940s and are associated with developmental and immune effects at exposure levels observed in the general population.

**Objective:**

To evaluate differences in exposure to 14 PFAS by sociodemographic factors among U.S. pregnant women.

**Methods:**

We combined maternal PFAS measurements from pregnant women from twelve pediatric cohorts drawn from the Environmental influences on Child Health Outcomes (ECHO) consortium’s public-use dataset (*n* = 3,043). Geometric means were estimated by race, ethnicity, and education. We used multivariable linear regression to estimate percent difference in log-transformed PFAS levels and logistic regression to assess odds of detectable PFAS levels by sociodemographic factors, adjusting for cohort, maternal age, trimester, year of sample collection, parity, BMI, fish consumption, and breastfeeding.

**Results:**

Compared with White mothers, Black mothers had lower levels of PFOA (−23%; 95% CI −29%, −16%), PFOS (−9%; 95% CI −16%, −1%), and PFHxS (−23%; 95% CI −29%, −14%) based on linear analysis, and lower detection of PFOSA, EtFOSAA, PFDoDA, and PFPeA and higher detection of PFBS and PFHxA based on logistic analysis. Compared with White mothers, Asian mothers had higher levels of PFNA ( + 26%; 95% CI 13%, 40%), PFDA ( + 60%, 95% CI 36%, 89%), and PFUnDA (+95%; 95% CI 63%, 134%) based on linear analysis and higher detection of PFPeA based on logistic regression. Greater weekly fish consumption was found to be a significant predictor of PFAS concentrations but did not attenuate associations with Asian race. Compared with non-Hispanic mothers, Hispanic mothers had lower levels of most of PFAS analytes studied. Higher levels of maternal education were associated with increased levels of most PFAS analytes.

**Significance:**

In data drawn from this US consortium, documents continued widespread PFAS exposure, with higher levels noted for certain racial groups, non-Hispanic ethnicity, and higher educational attainment.

**Impact statement:**

We analyzed data from a combined sample of U.S. pregnant women (*n* = 3043) in the ECHO public-use dataset to assess exposure to 14 per- and polyfluoroalkyl substances (PFAS), including both legacy and less-studied compounds. Higher levels of some PFAS were observed by race, ethnicity, and education. Our findings document continued widespread PFAS exposure in the U.S., including among fetuses and newborns who may be especially vulnerable to developmental and immune effects. Ongoing biomonitoring is critical for understanding changing exposure patterns across racial, ethnic, and socioeconomic groups.

## Introduction

Per-and polyfluoroalkyl substances (PFAS) are a large group of thousands of synthetic compounds characterized by their carbon-fluorine bond which is strong and difficult to break [1]. PFAS have been widely used in the manufacturing of commercial and industrial products, and numerous consumer goods since the 1940s [2, 3]. The unique chemical properties of PFAS make them resistant to extreme heat and chemical reactions – underpinning their utility for use in the manufacturing of commercial and industrial products and accounting for their extreme persistence in the environment [2]. They do not break down in the body [4], and several PFAS are eliminated slowly over time (half-lives of 1.5 years – 13 years) [5]. PFAS are detected in the blood serum of virtually all people in the United States (U.S.) [6], but serum concentrations of several long-chain PFAS have decreased in the general population [6] since large U.S. manufacturing phase-outs of PFOS (perfluorooctanesulfonic acid) and PFHxS (perfluorohexane sulfonic acid) in 2000–2002 [7] and PFOA (perfluorooctanoic acid) and PFNA (perfluorononanoic acid) in 2010 [8]. Health effects of PFAS such as decreased antibody response following vaccination, decreased fetal growth, and increased serum cholesterol, liver enzymes, and uric acid have been observed at exposure levels commonly observed in the general population [2].

Children are exposed to PFAS beginning *in-utero* and are born with PFAS concentrations strongly correlated to those of their mother [9]. Evaluating PFAS exposures in mothers is important for understanding how to reduce exposure *in-utero* [9] and postnatally through breast milk [10]. Concentrations of PFAS in breast milk of women from U.S. and Canada have been shown to exceed ATSDR drinking water Minimal Risk Levels [10, 11] and USEPA Maximum Contaminant Levels [12]. Given the long half-lives of many long-chain PFAS, levels measured in serum may reflect exposure that occurred years prior and cannot be disentangled from current or ongoing exposure. This is of particular interest among pregnant mothers whose PFAS exposure should be avoided well before pregnancy [13].

PFAS exposure observed in the U.S. general population is driven by dietary sources and consumer product use [14]. Dietary sources include fish and seafood, other animal products, and high-fat foods [15]. Future regulations and PFAS phase-outs are expected to reduce exposure to PFAS from intentional use in consumer and dietary sources including food-packaging [16]. However exposures from PFAS accumulation in our ocean and marine food chains [14], freshwater sources [17], and uptake of PFAS by plants and animals is likely to persist since environmental contamination with PFAS persists indefinitely. PFAS can bioaccumulate in fish and bioaccumulation potential varies by congener, fish species, and the water environment [18]. Consumption of fish and seafood, especially recreationally caught freshwater fish, may be a major source exposure to PFAS, particularly PFNA and PFUnDA [17, 19, 20]

PFAS exposures have been shown to vary across populations by sociodemographic factors [21, 22], and geography [21, 23]. Racial and ethnic differences in PFAS exposure have long been documented in the U.S. general population [24], including higher observed levels of certain PFAS among Asian Americans [25] thought to be from greater fish consumption [26]. However, few studies have characterized differences in PFAS exposures by sociodemographic characteristics in U.S. pregnant women, in particular. This gap is especially true for the less commonly measured PFAS. Given pregnancy and early life are critical windows of exposure for impacts on the developing child and differences in PFAS body-burdens among racial, ethnic, and socioeconomic groups have implications for identifying populations with disproportionate exposure to PFAS, we sought to address these gaps using a national US sample of pregnant mothers.

## Materials and methods

### Study population

The Environmental Influences on Child Health Outcomes (ECHO) program is a network of demographically and geographically diverse pediatric and pregnancy cohorts across the U.S. ECHO cohort sites have contributed extant data collected under parent protocols prior to the initiation of ECHO in 2016 and harmonized by the ECHO Data Analysis Center (DAC), as well as data collected under a common ECHO protocol. Data from ECHO are available in a deidentified publicly available dataset via the Data and Specimen Hub (DASH, dash.nichd.nih.gov/study/424643). A second release of ECHO-DASH data (released January 8, 2024) includes records for 32,311 pregnancies among 21,801 women and 30,904 children from 69 cohorts. All uses of the data were performed in accordance with the data use agreement; this study is classified as not human subjects research because of the de-identified and public-use nature of the data.

### PFAS measures

**Fig. 1:**
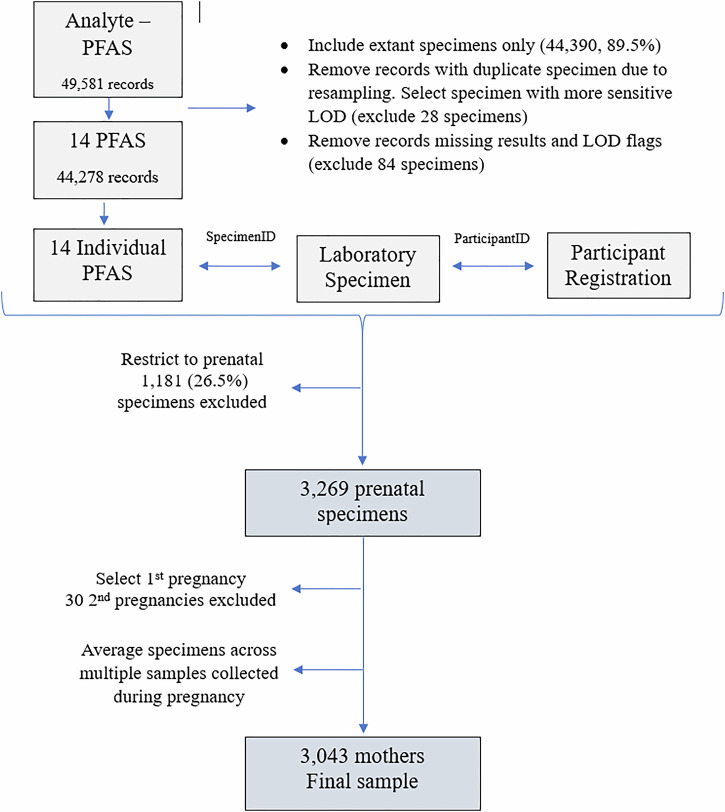
Process and workflow for identifying combined sample. Step-by-step process for deriving combined sample including inclusions, exclusions, and the resulting sample sizes at each stage.

Extant maternal PFAS measurements in serum collected during pregnancy were available from twelve ECHO cohorts. These PFAS data were combined (Fig. 1). Records missing all analytical results and flag indicator information indicating above and below the level of detection (LOD) were removed. Analytical results with the same PFAS analyte and specimen were deduplicated, and results with the lower, more sensitive, LOD were retained. PFAS specimens collected prenatally (trimesters 1, 2, or 3) were included. If PFAS data were collected from multiple trimesters, these analytical values were averaged and one trimester was randomly selected for inclusion as the indicator used in regression modeling. A weight (1 divided by the square root of the number of samples averaged), was calculated to account for difference in variance among values based on one sample versus multiple samples. When linear and branched PFOA or PFOS isomers were measured, these were summed as total PFOA or total PFOS.

Data were available for a total of fourteen PFAS (ng/mL) from among the 12 cohorts in which PFAS were analyzed in serum or plasma (Supplementary Table 1). Studies have demonstrated comparability between serum and plasma PFAS measures [27]. PFAS concentrations in serum or plasma samples were measured in several different laboratories including New York State’s Wadsworth Center, Centers for Disease Control and Prevention laboratories, California Department of Toxic Substances, Emory CHEAR lab, and EPA’s National Exposure Research Laboratory. Other studies have demonstrated the utility of these combined serum and plasma PFAS data [28, 29]. A total of seven PFAS for which ≥ 50% of sampled participants had measured levels above the LOD were included for further analysis as continuous variables: PFOA; PFOS; PFHxS; PFNA; 2-(N-Methyl-perfluorooctane sulfonamido) acetic acid (NMFOSAA); perfluorodecanoic acid (PFDA); and perfluoroundecanoic (PFUnDA). We also included a summed value for the four most commonly detected PFAS (PFOA, PFOS, PFHxS, PFNA) as an independent value.

For these continuous PFAS, truncated multiple imputation by chained equations was performed for PFAS concentrations below the LOD while considering race, ethnicity, maternal education, maternal age, cohort, parity, trimester of specimen collection, and year of sample collection [30]. Multiple imputation can reduce bias due to the unreliability of data below the LOD and is less biased than using simple imputation methods such as LOD/$\surd 2$ [31]. We set the cutoff for imputation, below which values may be drawn for imputation, using the maximum LOD across all samples defined as less than 0.1 ug/L for each of the seven PFAS imputed. For this procedure we used the “mice” package in R [30]. The “mice” package also imputes missing values for the categorial variables of interest (race, ethnicity, and maternal education), using the values of the other included variables to help inform the imputed values. We generated five imputed datasets which were combined to average into a single estimate accounting for the variability between the imputed datasets.

Seven PFAS for which levels were below the LOD in more than 50% of participants were included for further analysis as categorical variables: perfluorooctane sulfonamide (PFOSA); 2-(N-Ethyl-perfluorooctane sulfonamido) acetic acid (EtFOSAA); perfluoroheptanoic acid (PFHpA); perfluorododecanoic acid (PFDoDA); perfluorobutane sulfonate (PFBS); perfluorohexanoic acid (PFHxA); and perfluoro-n-pentanoic acid (PFPeA). Each of these seven PFAS were categorized into binary variables (less than LOD and greater than or equal to the LOD)

### Covariate and predictor information

The ECHO DAC undertook harmonization efforts to create comparable data across the cohorts, and harmonized variables were available for race, ethnicity, and maternal education. Additional covariates identified a priori included parity, trimester, year of sample collection, and maternal age [9, 28]. Maternal age at sample collection was calculated from date of birth and estimated delivery date, and where missing, maternal age was estimated from other survey components collected during pregnancy. BMI was considered as a covariate as it may be associated with PFAS concentrations and sociodemographic factors. Fish consumption was also considered, given that it is an important source of PFAS exposure that varies by the sociodemographic factors of interest (e.g. differing fish consumption across particular racial and ethnic groups). Maternal self-reported weekly fish consumption (# of fish meals per week) collected through dietary questionnaires administered during pregnancy were harmonized by ECHO to provide all comparable data in a single file. Ever breast feeding was also evaluated because it is an important factor in reducing maternal PFAS concentrations, and breastfeeding can vary by sociodemographic factors.

## Statistical analysis

Analyses were performed using SAS v 9.4 and RStudio v. 4.3.3. Spearman correlation coefficients were calculated between each pair of the seven continuous PFAS. The geometric mean and 95% confidence intervals (CI) along with percentile distribution (25^th^, 50^th^, 75^th^ and 95^th^) were calculated for each of the seven PFAS. We compared geometric means by year of sample collection categories to corresponding values for the U.S. general population as available from the National Health and Nutrition Examination Survey (NHANES) [32], restricted to women within the same age range as women in the ECHO combined sample (ages 16–47 years). Because the planned 2019–2020 NHANES cycle was interrupted due to COVID which resulted in a non-representative sample, it was not included for this comparison.

Seven PFAS analyzed as continuous data were log transformed and linearly regressed on race (White, Black, Asian and Other), ethnicity (non-Hispanic and Hispanic), maternal education (less than high school, high school graduate, some college, and Bachelor’s degree or greater), adjusting for cohort, year of sample collection, maternal age, trimester, and parity to estimate the adjusted percent change of PFAS serum concentration across levels of the independent variable of interest, relative to the reference level. Reference categories included White race, non-Hispanic ethnicity, less than high school education, and Cohort #10 as the largest of the cohorts with available data for each of the seven continuous PFAS. Weights to account for averaging of samples were included in linear regression. Additional co-control of PFOA and PFOS, the most highly correlated PFAS, was evaluated to determine how this impacted effect estimates. To reduce the impact of collinearity for correlated PFAS in the same model, PFAS covariates were coded as indicator variables for quartile categories. The earliest of the cohorts to collect samples (from 1998–2002) had the highest concentrations of PFOA, PFOS, and NMFOSAA concentrations among the sample. Due to the extreme values, we removed this cohort as a sensitivity analysis.

Distributions of the categorized PFAS ( < LOD or ≥LOD) were compared using chi-square test or Fisher’s exact test when counts were below five. For each of the categorized PFAS, logistic regression was used to estimate odds ratios (OR) and 95% confidence intervals (CI) for having levels ≥LOD in association with race, ethnicity, and education categories with increasing levels of adjustment for parity, trimester, year of sample collection and maternal age. Given the small number of individuals above the LOD for the categorized PFAS, additional co-control for correlated PFAS was not performed.

Additional analyses were performed to determine the impact of adjusting for BMI, fish consumption, or ever breast feeding on PFAS concentration across subgroups of race, ethnicity, and education. Subsample analyses were restricted to include individuals with available BMI data (82% of the data, includes *n* = 9–11 cohorts depending on PFAS congener), available maternal fish consumption data during pregnancy (46% of the data, includes *n* = 5–7 cohorts depending on PFAS congener), and yes/no ever breastfeeding (55% of the data, includes 8–10 cohorts depending on PFAS congener). Both maternal BMI and fish consumption were categorized into quartiles.

## Results

**Fig. 2:**
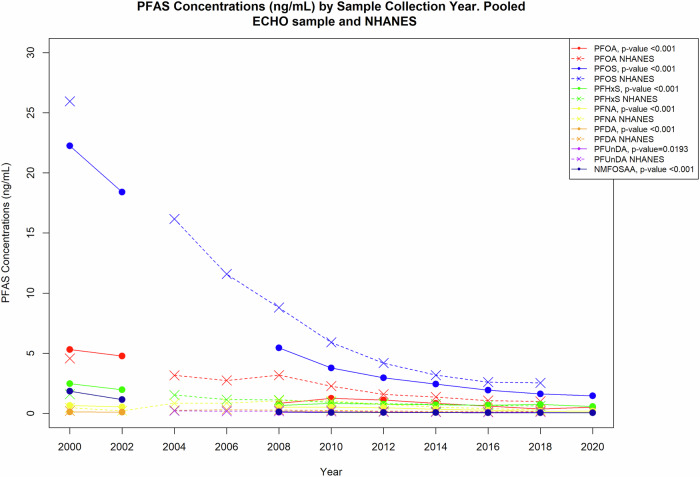
PFAS concentrations over time in the ECHO pooled sample (solid line) overlayed with NHANES (dotted line) sample restricted to women aged 16-47 years. Significance values for ECHO trend data were generated by regressing sample collection year as linear predictor of PFAS concentrations.

All pairwise Spearman correlations between pairs of seven PFAS analyzed continuously were statistically significant (*p*-value < 0.001) with the exception of PFUnDA with PFHxS and with NMFOSAA (Supplementary Table 2). The strongest correlations were between PFOA and PFOS (*p* = 0.804); and the weakest correlation was between PFHxS and PFUnDA (*p* = -0.0517). Sampling across parent cohorts occurred nonconsecutively from 1998 through 2020 (Fig. 2). Geometric means of the seven continuous PFAS in the combined ECHO sample by sample collection year categories are plotted together with geometric means of available PFAS from NHANES restricted to women aged 16–47 years (Fig. 2). Each of the seven PFAS had a statistically significantly decreasing trend with increasing sample year.

The distribution of covariates in the full sample of mothers (*n* = 3043) is available in Table 1. Mothers were mostly white, non-Hispanic, and the majority had a Bachelor’s degree or higher education. Each of the twelve cohorts represented between 1 and 18% of the total combined sample. Geometric mean PFAS concentrations varied among cohorts (Supplementary Table 3). This was the first child for 50% of mothers, second for 29% of mothers, and greater than second for 20% of mothers. The highest number of specimens were collected in the second trimester (41%).

**Table 1:** Characteristics of the ECHO-DASH study sample with PFAS data available, *n* = 3043.

Covariates	Categories	*n* (%)
**Race**	White	2031 (67%)
	Black	564 (19%)
	Asian	187 (6%)
	Other	106 (3%)
	Missing	155 (5%)
**Ethnicity**	Non-Hispanic	2442 (80%)
	Hispanic	588 (19%)
	Missing	13 (0%)
**Maternal Education**	Less than high school	202 (7%)
	High school	421 (14%)
	Some college	587 (29%)
	Bachelor’s degree	1778 (58%)
	Missing	55 (2%)
**Maternal age**	<25 years	444 (15%)
	25 - <30 years	697 (23%)
	30 - <35 years	1092 (36%)
	≥35 years	735 (24%)
	Missing	75 (2%)
**Parity**	1 child	1510 (50%)
	2 children	891 (29%)
	More than 2 children	595 (20%)
	Missing	47 (2%)
**Specimen Collection**	Trimester 1	837 (28%)
	Trimester 2	1236 (41%)
	Trimester 3	970 (32%)
**Year of sample collection**	1998–2000	434 (14%)
	2001–2002	118 (4%)
	2003–2004	0 (0%)
	2005–2006	0 (0%)
	2007–2008	27 (1%)
	2009–2010	487 (16%)
	2011–2012	441 (14%)
	2013–2014	358 (12%)
	2015–2016	537 (18%)
	2017–2019	498 (16%)
	2020–2021	43 (1%)
	Missing	100 (3%)
**Cohort**	Cohort−1	81 (3%)
	Cohort−2	271 (9%)
	Cohort-3	206 (7%)
	Cohort-4	236 (7%)
	Cohort-5	349 (11%)
	Cohort-6	552 (18%)
	Cohort-7	345 (11%)
	Cohort-8	34 (1%)
	Cohort-9	74 (2%)
	Cohort−10	390 (13%)
	Cohort−11	341 (11%)
	Cohort−12	164 (5%)

**Fig. 3:**
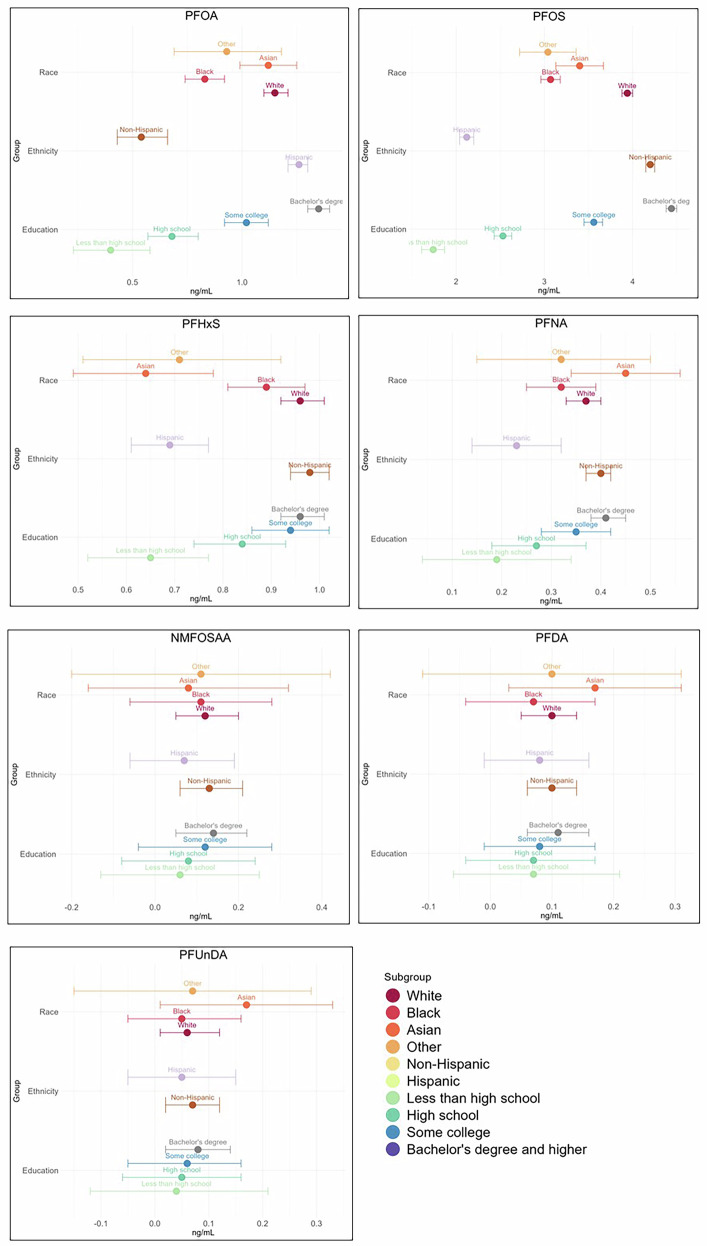
Geometric means and 95% confidence intervals for PFAS across sociodemographic categories. Panels show geometric means and 95% confidence intervals for each of seven PFAS by race, ethnicity, and education. Error bars represent 95% confidence intervals. Note: “Bachelor’s degree” includes Bachelor’s degree or higher.

Geometric means of the seven continuous PFAS by sociodemographic factors including race, ethnicity, and education are presented in Fig. 3. The highest geometric means for PFOA, PFOS, PFHxS, and NMFOSAA were among White race participants, whereas Asian race participants had the highest geometric means of PFNA, PFDA, and PFUnDA. Geometric means among participants of Hispanic ethnicity were lower than non-Hispanic for each of the seven PFAS. Geometric means increased with higher levels of educational attainment for each of the seven evaluated PFAS. Levels of all seven PFAS varied significantly across cohorts (Supplementary Table 3). The cohort with the earliest sample collection had the highest levels of PFOA, PFOS, and NMFOSAA.

Adjusted percent change in PFAS levels by race, ethnicity, and maternal education are presented in Table 2. Black race was significantly associated with lower levels of PFOA (−23%; 95% CI −29%, −16%); PFOS (−9%; 95% CI −16%, −1%); PFHxS (−23%;95% CI −29%, −14%); the sum of these three PFAS and PFNA (−11%; 95% CI −16%, -4%); and with non-statistically significant lower levels of PFNA compared to White race. Asian race was associated with lower levels of PFHxS (−25%; 95% CI -32%, −13%), and higher levels of in PFNA (26%; 95% CI 13%, 40%), PFDA (60%; 95% CI 36%, 89%), and PFUnDA (95%; 95% CI 63%, 134%) compared to White race. Following adjustment for PFOA in models predicting PFOS, lower levels were no longer observed among Black race compared with White race (1%; 95% CI −7%, 8%); but the association of Black race with lower PFOA remained after co-adjustment for PFOS (−20%, 95% CI −26%, −14%) (Supplementary Table 6). Mothers reporting other race had lower levels of PFHxS (−19%; 95% CI −28%, -5%) compared with White mothers. Unadjusted estimates of percent difference are provided in Supplementary Table 4–11 and generally show similar patterns as adjusted estimates but vary in magnitude and statistical significance.

**Table 2:** Percent change and 95% confidence interval of log-transformed PFAS by race, ethnicity, and maternal education.

	PFOA		PFOS		PFHxS		PFNA		∑PFAS		NMFOSAA		PFDA		PFUnDA	
Factors	% Δ	95% CI	% Δ	95% CI	% Δ	95% CI	% Δ	95% CI	% Δ	95% CI	% Δ	95% CI	% Δ	95% CI	% Δ	95% CI
**Race**																
White	----	----	----	----	----	----	----	----	----	----	----	----	----	----	----	----
Black	**−23%**	**−29%, −16%**	**-9%**	**−16%, −1%**	**−23%**	**−29%, −14%**	-5%	−12%, +3%	**−11%**	**−16%, -4%**	+1%	−13%, +17%	−12%	-52%, +50%	−1%	−22%, +26%
Asian	-0%	−11%, +11%	-0%	−10%, +11%	**−25%**	**-32%, −13%**	**+26%**	**+13%**, + **40%**	-3%	−11%, +6%	−15%	−29%, +1%	**+60%**	**+36%**, + **89%**	**+95%**	**+63%**, + **134%**
Other	−10%	−21%, +4%	−10%	−22%, +3%	**−19%**	**−28%, -5%**	-3%	−16%, +11%	-9%	−18%, +2%	+6%	−17%, +35%	+2%	−19%, +29%	-0%	−24%, +30%
**Ethnicity**																
Non-Hispanic	----	----	----	----	----	----	----	----	----	----	----	----	----	----	----	----
Hispanic	**-8%**	**−16%, −1%**	**−18%**	**−24%, −12%**	**−29%**	**-35%, −22%**	-4%	−10%, +5%	**−16%**	**−21%, −11%**	**−14%**	**−24%, -3%**	+2%	−11%, +17%	−14%	−29%, +5%
**Maternal Education**																
Less than HS	----	----	----	----	----	----	----	----	----	----	----	----	----	----	----	----
HS degree	+13%	−2%, +30%	+5%	-6%, +17%	+6%	-6%, +21%	+8%	-4%, +21%	+7%	−2%, +18%	-3%	−24%, +23%	-8%	-31%, +22%	-4%	-32%, +36%
Some college	**+24%**	**+7%**, + **43%**	**+17%**	**+5%**, + **31%**	**+17%**	**+3%**, + **32%**	**+20%**	**+7%**, + **35%**	**+19%**	**+8%**, + **30%**	-5%	−24%, +18%	+3%	−20%, +32%	+6%	−25%, +50%
Bachelor’s	**+18%**	**+1%**, + **37%**	**+17%**	**+4%**, + **30%**	+12%	−1%, +27%	**+24%**	**+11%**, + **39%**	**+17%**	**+5%**, + **29%**	-9%	-30%, +19%	+17%	-7%, +48%	+29%	-4%, +73%

Models adjusted for race, ethnicity, maternal education, cohort, parity, trimester, year of sample collection, and maternal age.

Bold indicates statistical significance alpha of 5%.

Hispanic ethnicity was associated with lower concentrations of several PFAS relative to non-Hispanics, including PFOA (-8%; 95% CI −16%, −1%), PFOS (−18%;95% CI −24%, −12%), PFHxS (−29%; 95% CI −35%, −22%), the sum of four PFAS (−16%; 95% CI −21%, −11%), and NMFOSAA (−14%; 95% CI −24%, −3%) (Table 2). Lower levels were also noted for concentrations of PFNA, PFDA, and PFUnDA based on Hispanic ethnicity, but these estimates were consistent with the null. Following co-adjustment for PFOS, non-Hispanic ethnicity was no longer associated with PFOA (Supplementary Table 4). Following co-adjustment for PFOA, the association of Hispanic ethnicity with lower levels of PFOS remained (−16%; 95 CI −21%, 10%) (Supplementary Table 5).

Greater maternal educational attainment (high school degree, some college, college degree or higher) was associated with higher levels of PFOA, PFOS, PFHxS, PFNA, PFDA, and PFUnDA compared with less than high school education (Fig. 2). For example, levels of PFOA increased 13% (95% CI −2%, 30%) among mothers reporting a high school degree, 24% (95% CI 7%, 43%) for some college, and 18% (95% CI 1%, 37%) for Bachelor’s degree or higher compared with mother’s reporting less than high school. Higher levels of maternal education were associated with lower levels of NMFOSAA. When PFOS was adjusted for PFOA, the observed associations with increasing levels of educational attainment were attenuated. In contrast, when the model predicting PFOA concentration was adjusted for PFOS, the association with maternal education was also attenuated but remained statistically significant among mothers with some college compared with less than high school (15%; 95% CI 1%, 31%) (Supplementary Table 4). Sensitivity analysis removing the cohort with earliest sample collection found the associations generally unchanged (Supplementary Tables 4–11).

In analyses including BMI, increasing levels of BMI were not statistically significantly associated with levels of PFAS. In our analyses adjusting for fish consumption, increasing weekly fish meals was associated with higher levels of PFNA, PFDA, and PFUnDA (Supplementary Tables 4–11). For example, levels of PFDA increased 8% (95% CI −17%, 40%) among mothers reporting 0.23- < 0.92 weekly fish meals, 26% (95% CI −5%, 66%) for 0.92−1.69 weekly fish meals, and 47% (95% CI 10%, 95%) for more than 1.69 meals compared with the lowest fish consumption category (0- < 0.23 weekly fish meals). Adjustment for weekly fish consumption did not attenuate associations between Asian race and increased levels of PFNA, PFDA, and PFUnDA, or associations noted for Black race compared with White race. Adjustment for fish consumption did attenuate some of the observed associations between maternal education and PFOA, PFOS, and PFNA, particularly at the highest level of educational attainment (Supplementary Tables 4−1^1^). For example, levels of PFOA increased 18% (95% CI 1%, 37%) among mothers with college education or more compared with less than high school and after adjustment for fish consumption, education was no longer associated with increased PFOA 5% (95% CI −13%, 27%). Adjusting for breast feeding attenuated increases in PFOS and in the sum of four PFAS among Black mothers compared with White mothers race;, decreases in PFOA and PFNA with Hispanic mothers compared with non-Hispanic mothers, and increases in PFOA, PFOS, PFHxS, PFNA, and the sum of four PFAS with maternal education at highest educational level compared with lowest (Supplementary Tables 4–11).

Count and percent of binary PFOSA, EtFOSAA, PFHpA, PFDoDA, PFBS, PFHxA, and PFPeA (<LOD, ≥LOD) are available in Supplementary Table 12. Adjusted odds ratios and 95% CI for the association with sociodemographic factors are provided in Table 3, and results from models further adjusted for race, ethnicity, maternal education, maternal BMI, and fish consumption are available in Supplementary Table 13. Compared with White race, Black race participants were less likely to have serum levels ≥LOD for PFOSA (OR = 0.43; 95% CI 0.21, 0.88), EtFOSAA (OR = 0.53; 95% CI 0.28, 0.97), PFDoDA (OR = 0.36; 95% CI 0.14, 0.93), and PFPeA (OR = 0.34; 95% CI 0.19, 0.60). In contrast, compared with White race, mothers reporting Black race were more likely to have serum levels ≥LOD for PFBS (OR = 3.10; 95% CI 1.71, 5.63) and PFHxA (OR = 12.38; 95% CI 4.62, 33.19). When compared to White race, mothers reporting Asian race were more likely to have serum levels ≥LOD for PFDoDA (OR = 1.84; 95% CI 0.97, 3.51) and PFPeA (OR = 8.87, 95% CI 2.01, 39.07). Hispanic ethnicity was associated with being less likely to have serum levels ≥LOD for EtFOSAA, PFHpA, PFBS, PFHxA compared with non-Hispanic ethnicity and more likely to be above the LOD for PFPeA. Higher maternal educational attainment compared with those who had a high school degree or less was associated with increased odds of PFOSA ≥ LOD. Generally, additional adjustment did not change estimates although several associations were no longer statistically significant, although confidence intervals were especially wide in fully adjusted models (Supplementary Table 13).

**Table 3:** Odds ratio (OR) and 95% Confidence Intervals (CIs) of PFAS serum level ≥ LOD and sociodemographic factors unadjusted (crude) and adjusted for year of sample collection, maternal age, parity and trimester.

		Race White *Reference*			Ethnicity non-Hispanic *Reference*	Maternal education High school or less *Reference*	
		Black OR (95% CI)	Asian OR (95% CI)	Other OR (95% CI)	Hispanic OR (95% CI)	Some college OR (95% CI)	College OR (95% CI)
**PFOSA**	Crude	**0.33 (0.17-0.66)**	0.62 (0.27−1.44)	0.50 (0.15−1.61)	**0.32 (0.16-0.64)**	**7.19 (2.12−24.39)**	**10.74 (3.39-34.04)**
	Adjusted	**0.43 (0.21-0.88)**	0.69 (0.29−1.62)	0.57 (0.18−1.87)	0.51 (0.25−1.04)	**5.39 (1.56−18.63)**	**7.52 (2.25−25.20)**
**EtFOSAA**	Crude	**0.59 (0.45-0.77)**	**0.57 (0.39-0.85)**	0.66 **(**0.40−1.10)	**0.49 (0.36-0.68)**	**2.35 (1.57-3.52)**	**3.36 (2.37-4.77)**
	Adjusted	**0.53 (0.28-0.97)**	0.96 **(**0.48−1.92)	1.11 **(**0.43−2.88)	**0.51 (0.26-0.97)**	1.02 **(**0.48−2.15)	1.60 **(**0.82-3.12)
**PFHpA**	Crude	0.85 **(**0.55−1.29)	1.30 **(**0.76−2.24)	1.48 **(**0.74−2.93)	**0.38 (0.23-0.61)**	1.06 **(**0.60−1.90)	**1.98 (1.30-3.02)**
	Adjusted	1.12 **(**0.69−1.83)	1.11 **(**0.63−1.95)	1.57 **(**0.78-3.15)	**0.38 (0.22-0.66)**	1.09 **(**0.60−1.99)	1.58 **(**0.94−2.65)
**PFDoDA**	Crude	**0.44 (0.21-0.94)**	**2.15 (1.19-3.86)**	0.93 **(**0.32−2.65)	**2.15 (1.23-3.76)**	1.19 **(**0.58−2.43)	0.81 **(**0.44−1.48)
	Adjusted	**0.36 (0.14-0.93)**	1.84 **(**0.97-3.51)	0.74 **(**0.25−2.23)	1.66 **(**0.90-3.05)	1.08 **(**0.49−2.40)	0.75 **(**0.36−1.54)
**PFBS**	Crude	**3.54 (2.11-5.95)**	0.99 **(**0.29-3.31)	1.03 **(**0.24-4.42)	**0.43 (0.20-0.92)**	0.56 **(**0.27−1.16)	**0.47 (0.27-0.81)**
	Adjusted	**3.10 (1.71-5.63)**	0.71 **(**0.16-3.08)	1.06 **(**0.25-4.59)	**0.25 (0.11-0.59)**	0.64 **(**0.31−1.35)	0.77 **(**0.39−1.52)
**PFHxA**	Crude	**16.75 (7.09-39.58)**	0.86 **(**0.01-7.39)	-----	**0.23 (0.07-0.77)**	0.64 **(**0.35−1.15)	**0.21 (0.11-0.38)**
	Adjusted	**12.38 (4.62-33.19)**	1.23 **(**0.14−11.05)	-----	**0.39 (0.11−1.37)**	0.79 **(**0.39−1.58)	**0.37 (0.17-0.78)**
**PFPeA**	Crude	**0.41 (0.27-0.63)**	**7.75 (1.79-33.51)**	1.25 **(**0.46-3.39)	**4.88 (2.14−11.13)**	1.47 **(**0.87−2.50)	1.37 **(**0.89−2.10)
	Adjusted	**0.34 (0.19-0.60)**	**8.87 (2.01-39.07)**	1.46 **(**0.51-4.20)	**5.15 (2.19−12.11)**	1.08 **(**0.60−1.93)	0.74 **(**0.43−1.29)

Bold indicates statistical significance alpha of 5%.

## Discussion

This study included a demographically and geographically diverse sample of twelve combined U.S. pediatric and pregnancy cohorts from across the U.S. Maternal PFAS plasma or serum concentrations were available from 1999–2020 and decreases in legacy PFAS over time were observed in this U.S. sample of pregnant women. Observed decreases are likely due to reductions in U.S. manufacturing from a series of phase-outs to eliminate PFOA, PFOS, PFNA and PFHxS by 2015 [7, 8]. Our study found that PFAS concentrations differed substantially by race, ethnicity, and education.

Out of the 14 PFAS evaluated in this study, mothers of Black race had lower levels of PFOA, PFOS, PFHxS, the sum of four PFAS, PFOSA, EtFOSAA, PFDoDA, and PFPeA and higher levels of PFBS and PFHxA, compared with White mothers. Race has been identified as an important correlate of PFAS exposure [22, 23, 33], including among pregnant women in the U.S. [34, 35]. Consistent with observations in this study, a study of a cohort of pregnant women living in Cincinnati, Ohio (not an ECHO cohort) also found that non-Hispanic Black women had lower serum concentrations of PFOA, PFOS, PFHxS, and PFNA compared with White women [36]. Lower concentrations of PFOA, PFOS, PFHxS, PFNA, and PFDA were also observed among Black Americans in a U.S. population-based study [25].

Our study found that Asian mothers had higher levels of PFNA, PFDA, PFUnDA, and PFPeA compared with mothers reporting White race. Levels of PFNA and PFDA among individuals of Asian race were also found to be higher than White individuals in a U.S. population study [25], and a study in midlife women found higher PFNA concentrations among Asian American women compared to White [23]. Higher observed levels of certain PFAS among Asian Americans have been thought to result from their greater fish consumption [26, 37]. We found that increased fish consumption was associated with higher levels of PFNA, PFDA, and PFUnDA, and adjusting for fish consumption did not attenuate the higher levels observed among Asian mothers. Despite increased exposure to some PFAS from fish consumption, health benefits associated with fish consumption may counter negative effects of PFAS [38], and this should be considered when developing dietary guidance for pregnant women.

Hispanic ethnicity was associated with lower levels of PFOA, PFOS, PFHxS, the sum of four PFAS, NMFOSAA, EtFOSAA, PFHpA, PFBS, and PFHxA and increased levels of PFPeA compared with non-Hispanic ethnicity. Hispanic ethnicity has also been shown to be an important predictor of lower PFAS exposure [22], including specifically among Mexican-Americans compared with non-Hispanic Whites [22, 25]. Lower PFAS levels are also reported among Hispanics born outside of the United States [22, 23]; our study was not able to evaluate country of birth.

Higher levels of maternal education were associated with higher serum levels of PFOA, PFOS, PFHxS, PFNA, and the sum of four PFAS as well as higher exposure to PFOSA and lower exposure to PFHxA compared to those with less than high school level of education. Higher education has been previously associated with higher levels of PFAS [22, 23, 39], including among a cohort of Chinese pregnant women [21]. Relatedly, income has been demonstrated to be an important predictor of PFAS concentration [22, 36], including among U.S. pregnant women from seven county-level cohort locations across the country [34]. Higher educational attainment and increased income are closely correlated which may explain, at least in part, the observed association with increased PFAS concentrations among those with the highest educational attainment in this study. However, maternal education may also impact dietary and consumer product use, despite income. Indeed, we observed increases in several PFAS associated with college education were attenuated when adjusted for weekly fish consumption. We also found that adjustment for breastfeeding attenuated increases in PFOA, PFOS, PFHxS, PFNA, and the sum of four PFAS with higher maternal educational attainment. Maternal education is strongly linked with breastfeeding rates and duration [40], and also results in decreased maternal body burdens of PFAS (and higher body burdens among infants), suggesting breastfeeding plays a mediating role in observed relationships with maternal education and PFAS exposure.

Factors that result in differences in PFAS concentrations among racial, ethnic, and socioeconomic groups are not well characterized [39] but could include differences in diet and use of consumer goods [26, 34, 41] and differences in local sources of PFAS contamination [34]. Dietary and consumer use patterns vary by sociodemographic factors, and diet may account for 10 to 20% of variation in PFAS exposure [42]. Fish intake, fast food consumption, microwave popcorn are several identified dietary sources of PFAS [23, 34, 36]; while beans, vegetable and fruit consumption have been associated with decreased PFAS levels [21, 43]. Sources of exposure to PFAS can include both food and food-packaging [44]. The US Food and Drug Administration announced that manufacturers had voluntarily agreed to end the use of grease-proof packaging that contains PFAS, although it may take several more years before old supplies are expended [45]. Personal care products [46] and consumer products such as use of stain repellants [36] have also been associated with higher PFAS serum concentrations, and patterns of personal care and consumer products use may also vary among racial, ethnic, and economic groups.

Geographic location is an important determinant of PFAS exposure [23]. Regional differences could be in part explained by contaminated drinking water sources [34, 47]. For example, former or current U.S. military installations, industrial sites, and wastewater treatment facilities have also been identified as sources of PFAS contamination of nearby water systems and wells [48]. USEPA drinking water standards have been finalized for six PFAS [12], and their implementation will likely lead to further reduction in PFAS exposure, particularly given that even low levels of PFAS in drinking water can have a large impact on body burdens [2]. Indeed, reduced PFAS body burdens are observed in communities which have reduced PFAS levels in contaminated drinking water sources [5]. In addition to contaminated drinking water, exposure to PFAS in some communities may be compounded by higher exposures from local agricultural products due to use of PFAS contaminated biosolids used on farmland [49]. A more detailed locational description of each cohort would allow the opportunity to evaluate spatial proximity to sources of PFAS contamination, but this information is unavailable in the public-use, deidentified ECHO-wide dataset.

Few studies have evaluated sociodemographic differences among concentrations of several of the less-studied PFAS evaluated in this study, including both longer-chain and shorter-chain PFAS. In our study we found longer-chain PFAS including PFOSA, EtFOSAA, PFHpA, and PFDoDA and shorter-chain PFAS including PFBS, PFHxA, and PFPeA were associated with categories of maternal race, ethnicity, and education. Long-chain perfluoroalkyl acids (PFAAs), defined as perfluoroalkyl carboxylates (those having a carboxylate functional group) having 8 or more carbons (e.g., PFOA, PFNA) and perfluoroalkyl sulfonates (those having a sulfonate functional group) with 6 or more carbons (e.g., PFHxS, PFOS [50]), have half-lives of several years (Supplementary Table 1) and have been phased out due to concerns about their biological persistence and potential health effects (Post, Gleason et al. 2017). In their place, alternatives including short-chain PFAAs and other types of PFAS with fewer carbons are increasingly used [2]. These shorter-chain PFAS generally have shorter half-lives (e.g., PFBS: 35 days, PFHxA: 32 days [51]), which, all other things being equal, are expected to result in lower health risks [52]. While we were not able to identify a human half-life estimate for several of the PFAS included in our study, (Supplementary Table 1), we may anticipate that the half-lives of those with long-chain lengths could potentially be several years. As long-chain PFAAs are phased-out, the importance of biomonitoring for alternative PFAS will be integral for understanding our human exposure burden [25, 53].

Our study has some limitations. Samples from different cohorts were not analyzed with the same PFAS laboratory analytical protocols, and the combined dataset includes samples collected throughout pregnancy trimesters and at birth. Given PFAS concentrations are dynamic throughout pregnancy and impact of trimester of sample collection on PFAS concentration varies by PFAS congener [9], which could lead to issues of comparability across the cohorts. To account for any impact of these differences, trimester of sample collection was included in multiple imputation models used to estimate values below the LOD, and we adjusted for trimester and cohort in multivariable models.

Additional analyses of dietary survey data collected during pregnancy were limited to fish consumption in this study. Future studies evaluating source of fish (marine, freshwater, or recreationally caught) and species of fish are warranted to contribute to guidance for women who are pregnant or plan on becoming pregnant on choice of fish to include in their diets. Additional analysis of other dietary sources and use of consumer products are also warranted for understanding sources of PFAS exposure to pregnant women. Although this analysis provides some information on several less-commonly studied PFAS (including PFHpA, PFHxA, PFPeA), the low detection frequency of these PFAS limits our comparisons of exposure levels among different sociodemographic groups.

## Conclusion

The ubiquitous detection of PFAS in blood serum indicates that exposure to PFAS has been widespread nationally in the U.S., including among fetuses and newborns who may be particularly vulnerable to developmental and immune effects from PFAS exposure. As changes in the use and release of PFAS to the environment occur, including through development of regulatory limits and use of alternatives, biomonitoring of PFAS is invaluable for understanding our changing exposure patterns, including among racial, ethic, and socioeconomic groups. In data drawn from a large US consortium, our work documents continued widespread PFAS exposure, with higher levels of some PFAS noted for certain racial groups, non-Hispanic ethnicity, higher educational attainment, and with increased fish intake. Further work is warranted for evaluating dietary and consumer product determinants of PFAS exposure, especially among pregnant women, to promote reduced PFAS exposures among fetuses and young children.

## Supplementary information

Supplementary Table1

Supplementary Table2

Supplementary Table3

Supplementary Table4

Supplementary Table5

Supplementary Table6

Supplementary Table7

Supplementary Table8

Supplementary Table9

Supplementary Table10

Supplementary Table11

Supplementary Table12

Supplementary Table13

## Data Availability

Data from ECHO are available in a deidentified publicly available dataset which can be request via the Data and Biospecimen Sharing Hub (DASH, dash.nichd.nih.gov/study/424643).
