# Supplementary material for: Sociodemographic predictors of PFAS exposure among a combined sample of U.S. pregnant women: an Environmental influences on Child Health Outcomes (ECHO) public-use dataset analysis
Source: J Expo Sci Environ Epidemiol. 2025 Dec 15;36(3):459–68. doi: 10.1038/s41370-025-00833-8 (PMC13143815; doi:10.1038/s41370-025-00833-8)
Supplement: Supplementary file 1 — Supplementary Table1 [file 41370_2025_833_MOESM1_ESM.pdf]

Supplemental Table 1. Available extant maternal PFAS data in ECHO collected during pregnancy; pooled from twelve U.S. cohorts

Acronym	Name<br>CAS Number	Chemical Structure	Carbon Chain Length	Half-life in humans (published values)	# of cohorts	Sample Size	% < LOD
PFOA	Perfluorooctanoic acid<br>**335-676-1**	$C_7F_{15}COOH$	8	1.5 – 5.1 years[1,2]	12	3043	3.1%
PFOS	Perfluorooctanesulfonic acid<br>**1763-23-1**	$C_8F_{17}SO_3$-	8	3.4 – 5.7 years[1,2,3]	12	3043	0.4%
PFHxS	Perfluorohexane sulfonic acid<br>**355-46-4**	$C_6F^{13}SO_3H$	6	2.8 – 8.5 years[1,2,3]	12	3043	0.7%
PFNA	Perfluorononanoic acid<br>**375-95-1**	$C_8F_{17}COOH$	9	1.7 – 3.5 years[3,4,5]	12	3042	3.2%
NMFOSAA	2-(N-Methyl-perfluorooctane sulfonamido) acetic acid<br>**2355-31-9**	$C_8F_{17}SO_2N(CH_3)$ CH_2COOH	8	Data not identified	9	2626	42.3%
PFDA	Perfluorodecanoic acid<br>**335-76-2**	$C_9F_{19}COO$	10	4 – 7.1 years[3,5]	11	2594	33.2%
PFUnDA	Perfluroundecanoic acid<br>**2058-94-8**	$C_{10}F_{21}COO$-	11	12.0 years[4]	9	1724	44.3%
PFOSA	Perfluorooctane sulfonamide<br>**754-91-6**	$C_8F_{17}SO_2NH_2$	8	Data not identified	9	2109	94.0%
EtFOSAA	2-(N-Ethyl-perfluorooctane sulfonamido) acetic acid<br>**2991-50-6**	$C_8F_{17}SO_2N(C_2H_5$ $)CH_2COOH$	8	Data not identified	8	1979	67.6%
PFHpA	Perfluoroheptanoic acid<br>**375-85-9**	$C_6F_{13}COOH$	7	130 days – 1.0 year[3,5]	7	1289	86.4%
PFDoDA	Perfluorododecanoic acid<br>**307-55-1**	$C_{11}F_{23}COO$-	12	Data not identified	8	1103	92.6%
PFBS	Perfluorobutanesulfonic acid<br>**375-73-5**	$C_4F_9SO_3H$	4	35 days[3]	8	1197	94.5%
PFHxA	Perfluorohexanoic acid<br>**307-24-4**	$C_5F_{11}COOH$	6	32 days[3]	4	439	80.0%
PFPeA	Perfluoro-n-pentanoic acid<br>**2706-90-3**	C_4F_9COOH	5	Data not identified	4	439	41.5%

Footnotes:
Source of half-life ranges
1. Li et al., (5); 2. Rosato et al., (50); 3. Dawson et al., (47); 4. Yu et al., (51); 5. Zhang et al., (52)
