# Supplementary material for: Sociodemographic predictors of PFAS exposure among a combined sample of U.S. pregnant women: an Environmental influences on Child Health Outcomes (ECHO) public-use dataset analysis
Source: J Expo Sci Environ Epidemiol. 2025 Dec 15;36(3):459–68. doi: 10.1038/s41370-025-00833-8 (PMC13143815; doi:10.1038/s41370-025-00833-8)
Supplement: Supplementary file 2 — Supplementary Table2 [file 41370_2025_833_MOESM2_ESM.pdf]

Supplemental Table 2. Spearman correlation coefficients between pairs of PFAS concentrations

	PFOA_RESULT4	PFOS_RESULT4	PFHXS_RESULT3	PFNA_RESULT3	NMFOSAA_RESULT3	PFDA_RESULT3	PFUNDA_RESULT3
	1	0.80477	0.54167	0.76046	0.76001	0.6548	0.33583
		**<.0001**	**<.0001**	**<.0001**	**<.0001**	**<.0001**	**<.0001**
PFOA_RESULT4	2948	2941	2932	2911	1492	1701	958
	0.80477	1	0.60423	0.71444	0.79577	0.59577	0.37498
	**<.0001**		**<.0001**	**<.0001**	**<.0001**	**<.0001**	**<.0001**
PFOS_RESULT4	2941	3029	3015	2938	1496	1733	961
	0.54167	0.60423	1	0.38338	0.6195	0.1931	-0.0517
	**<.0001**	**<.0001**		**<.0001**	**<.0001**	**<.0001**	0.1092
PFHXS_RESULT3	2932	3015	3022	2931	1491	1733	961
	0.76046	0.71444	0.38338	1	0.56588	0.71665	0.52576
	**<.0001**	**<.0001**	**<.0001**		**<.0001**	**<.0001**	**<.0001**
PFNA_RESULT3	2911	2938	2931	2945	1481	1710	960
	0.76001	0.79577	0.6195	0.56588	1	0.47241	0.06368
	**<.0001**	**<.0001**	**<.0001**	**<.0001**		**<.0001**	0.1447
NMFOSAA_RESULT3	1492	1496	1491	1481	1515	942	526
	0.6548	0.59577	0.1931	0.71665	0.47241	1	0.60631
	**<.0001**	**<.0001**	**<.0001**	**<.0001**	**<.0001**		**<.0001**
PFDA_RESULT3	1701	1733	1733	1710	942	1733	818
	0.33583	0.37498	-0.0517	0.52576	0.06368	0.60631	1
	**<.0001**	**<.0001**	0.1092	**<.0001**	0.1447	**<.0001**	
PFUNDA_RESULT3	958	961	961	960	526	818	961

Correlation categories	Pearson	Color coding
Strong	0.7-1	
Moderate	**0.3-0.69**	
Weak	0-0.29	
