# Supplementary material for: Sociodemographic predictors of PFAS exposure among a combined sample of U.S. pregnant women: an Environmental influences on Child Health Outcomes (ECHO) public-use dataset analysis
Source: J Expo Sci Environ Epidemiol. 2025 Dec 15;36(3):459–68. doi: 10.1038/s41370-025-00833-8 (PMC13143815; doi:10.1038/s41370-025-00833-8)
Supplement: Supplementary file 3 — Supplementary Table3 [file 41370_2025_833_MOESM3_ESM.pdf]

Supplemental Table 3 (PFOA, PFOS, PFHxS, PFNA). Years of sample collection, geometric means and standard errors and adjusted percent difference

			PFOA					PFOS					PFHxS					PFNA				
Cohort	n(%)	Years of Sample Collection	GM (ng/mL)	SE	% diff	95% CI		GM (ng/mL)	SE	% diff	95% CI		GM (ng/mL)	SE	% diff	95% CI		GM (ng/mL	SE	% diff	95% CI	
Overall	3043 (100	1998-2020	1.068737	0.021943				3.682199	0.021485				0.91516	0.017934				0.358913	0.015783			
Cohort-1	81 (3%)	2013-2017	0.651316	0.073784	1%	18%	24%	2.386547	0.055121	29%	7%	56%	1.255824	0.043024	217%	154%	294%	0.356088	0.057547	28%	5%	56%
Cohort-2	271 (9%)	2008-2013	1.400797	0.029916	81%	40%	133%	4.300815	0.032641	99%	54%	158%	0.77062	0.043212	1%	21%	30%	0.641401	0.031822	102%	40%	191%
Cohort-3	206 (7%)	2013-2018	0.644526	0.059064	33%	10%	62%	1.92198	0.055653	10%	6%	29%	0.967551	0.055404	169%	122%	225%	0.251534	0.057355	13%	5%	33%
Cohort-4	236 (7%)	2015-2018	0.145584	0.084853	-71%	-76%	66%	1.312635	0.038103	-10%	-22%	4%	1.090095	0.029244	226%	176%	285%	0.090703	0.068591	-55%	-62%	47%
Cohort-5	349 (11%)	2009-2011	1.256011	0.029618	93%	44%	160%	3.742893	0.026195	96%	45%	165%	0.864788	0.043655	37%	2%	84%	0.482499	0.029573	59%	4%	142%
Cohort-6	552 (18%)	1998-2002	5.416662	0.022863	245%	64%	622%	24.22579	0.09226	494%	189%	1123%	2.365006	0.036096	98%	3%	281%	0.639483	0.027471	-20%	-71%	125%
Cohort-7	345 (11%)	2009-2014	1.100984	0.037068	43%	14%	78%	2.382572	0.04829	11%	10%	38%	0.78149	0.045013	17%	6%	45%	0.414311	0.034366	30%	4%	76%
Cohort-8	34 (1%)	2006-2010	0.52735	0.270117	-27%	-51%	9%	4.446323	0.160643	94%	29%	192%	0.596249	0.214481	-8%	-36%	31%	0.556411	0.177981	60%	7%	176%
Cohort-9	74 (2%)	2008-2015	0.884879	0.061678	50%	14%	97%	2.82425	0.066136	55%	21%	100%	0.454932	0.074342	-5%	-27%	23%	0.477047	0.052192	71%	22%	141%
Cohort-10	390 (13%)	2013-2020	0.699335	0.037967	----	----	----	2.086319	0.041177	----	----	----	0.539092	0.046064	----	----	----	0.270655	0.032642	----	----	----
Cohort-11	341 (11%)	2013-2019	0.692127	0.039216	-6%	-16%	6%	1.712709	0.040147	-10%	-19%	1%	0.337181	0.04784	-30%	-38%	20%	0.259211	0.037833	-2%	-13%	10%
Cohort-12	164 (5%)	2015-2019	0.613088	0.047749	13%	2%	31%	2.608257	0.038337	52%	32%	76%	1.815157	0.031984	335%	269%	411%	0.267345	0.045245	25%	6%	48%

Supplemental Table 3 (NMFOSAA, PFDA, PFUNDA). Years of sample collection, geometric means and 95% confidence interval and adjusted percent difference

Cohort	n(%)	Years of Sample Collection	Sum of 4 PFAS					NMFOSAA					PFDA					PFuNDA				
			GM (ng/mL)	SE	% diff	95% CI		GM (ng/mL)	SE	% diff	95% CI		GM (ng/mL)	SE	% diff	95% CI		GM (ng/mL)	SE	% diff	95% CI	
Overall	3043 (100	1998-2020	0.392224	0.180544									0.096735	0.019929				0.066856	0.023542			
Cohort-1	81 (3%)	2013-2017	0.641401	0.031822	42%	21%	66%	0.117425	0.033643	-30%	-50%	2%	0.084785	0.076816	-4%	-27%	27%	0.106184	0.091044	68%	26%	123%
Cohort-2	271 (9%)	2008-2013	0.251534	0.057355	61%	20%	115%	0.076501	0.068835	20%	7%	54%	0.154391	0.046539	75%	22%	153%	0.076122	0.065879	16%	13%	56%
Cohort-3	206 (7%)	2013-2018	0.090703	0.068591	36%	19%	55%	0.052611	0.079052	-23%	-41%	0%	0.065518	0.079647	18%	10%	54%	0.052942	0.07018	13%	25%	70%
Cohort-4	236 (7%)	2015-2018	0.482499	0.029573	8%	5%	21%	0.042184	0.054623	-20%	-38%	2%	0.063671	0.054807	4%	23%	40%	0.040776	0.062226	-4%	-26%	25%
Cohort-5	349 (11%)	2009-2011	0.639483	0.027471	60%	14%	126%			0%	0%	0%										
Cohort-6	552 (18%)	1998-2002	0.414311	0.034366	273%	57%	787%	1.808652	0.028876	1854%	1069%	3166%	0.10497	0.052604	-39%	-78%	74%					
Cohort-7	345 (11%)	2009-2014	0.556411	0.177981	13%	12%	45%	0.060822	0.059519	-7%	-26%	17%	0.11019	0.055934	30%	1%	70%					
Cohort-8	34 (1%)	2006-2010	0.477047	0.052192	54%	1%	137%			0%	0%	0%						0.068436	0.241956	15%	32%	96%
Cohort-9	74 (2%)	2008-2015	0.270655	0.032642	34%	2%	76%	0.073502	0.110955	23%	10%	68%	0.109905	0.096352	33%	3%	83%	0.071242	0.116238	21%	17%	76%
Cohort-10	390 (13%)	2013-2020			----	----	----			----	----	----	0.081505	0.046348	----	----	----	0.059914	0.043057	----	----	----
Cohort-11	341 (11%)	2013-2019	0.259211	0.037833	-81%	58%	106%	0.045533	0.045151	-10%	-24%	-8%	0.127216	0.046827	51%	26%	80%	0.117021	0.048298	87%	57%	123%
Cohort-12	164 (5%)	2015-2019	0.267345	0.045245	0%	0%	0%	0.047228	0.067356	-17%	-37%	-8%	0.062516	0.065111	-1%	-26%	32%	0.054073	0.056757	6%	18%	35%
