# Supplementary material for: Sociodemographic predictors of PFAS exposure among a combined sample of U.S. pregnant women: an Environmental influences on Child Health Outcomes (ECHO) public-use dataset analysis
Source: J Expo Sci Environ Epidemiol. 2025 Dec 15;36(3):459–68. doi: 10.1038/s41370-025-00833-8 (PMC13143815; doi:10.1038/s41370-025-00833-8)
Supplement: Supplementary file 4 — Supplementary Table4 [file 41370_2025_833_MOESM4_ESM.pdf]

Supplemental Table 4: PFOA, includes estimated percent difference adjusted for race, ethnicity, education, cohort, parity, trimester, maternal age, and year of sample collection and 95% interval for final model, model adjusted for quartile of PFOS, model with Cohort #6 restricted, model adjusted for BMI, breast feeding, and weekly fish consumption

Mean (SD)		PFOA n=15,215			PFOA; adjusted for quartiles of PFOS (sensitivity analysis) n=15,215			PFOA; no AAU01 cohort (sensitivity analysis) n=12,455			PFOA (including BMI) n=12,910			PFOA (including breastfeeding) n=7,572			PFOA (including FISH) n=6,870			PFOA (unadjusted) n=15,125		
		%change	95 % CI		%change	95 % CI		%change	95 % CI		%change	95 % CI		%change	95 % CI		%change	95 % CI		%change	95 % CI	
Race																						
1	White	----			----			----			----			----			----			----		
2	Black	-23%	-29%	-16%	-20%	-26%	-14%	-25%	-32%	-16%	-26%	-33%	-18%	-27%	-40%	-11%	-20%	28%	-12%	-28%	-36%	-20%
3	Asian	0%	11%	11%	0%	-10%	10%	3%	-9%	17%	2%	-10%	16%	14%	-3%	35%	-6%	-19%	9%	-3%	-19%	17%
4	Other	-10%	21%	4%	-6%	-17%	6%	-12%	-26%	3%	-7%	-21%	8%	-14%	-29%	4%	-14%	-29%	4%	-19%	-36%	3%
Ethnicity																						
0	Non-Hispanic	----			----			----			----			----			----			----		
1	Hispanic	-	-16%	-1%	0%	-7%	8%	-9%	-17%	-1%	-9%	-18%	0%	-8%	-20%	5%	-4%	-13%	6%	-57%	-61%	-52%
Maternal education																						
1	Less than high sch	----			----			----			----			----			----			----		
2	High school degre	13%	-2%	30%	10%	-3%	25%	12%	-4%	30%	10%	-8%	31%	21%	-6%	55%	2%	-14%	22%	69%	36%	110%
3	Some college	24%	7%	43%	15%	1%	31%	18%	2%	37%	19%	0%	41%	28%	0%	64%	25%	-4%	49%	153%	107%	210%
4	Bachelor's degree	18%	1%	37%	10%	-4%	26%	18%	0%	39%	14%	-6%	37%	26%	-1%	60%	5%	-13%	27%	234%	176%	305%
Cohort																						
1	AAA01	1%	-18%	24%	-10%	-25%	8%	1%	-19%	26%	0%	-20%	24%	10%	-13%	39%	17%	-27%	89%			
2	AAF01	81%	40%	133%	35%	16%	57%	81%	38%	137%	80%	39%	133%	93%	43%	161%						
3	AAG01	33%	10%	62%	28%	8%	52%	41%	15%	73%	31%	4%	65%	75%	32%	131%	46%	-10%	136%			
4	AAP01	-71%	-76%	-66%	-69%	-73%	-63%	-71%	-75%	-65%	-72%	-76%	-66%	-66%	-76%	-53%						
5	AAS01	93%	44%	160%	-44%	21%	70%	99%	46%	172%				82%	-18%	304%	63%	-14%	210%			
6	AAU01	245%	64%	622%	166%	83%	287%				244%	61%	634%	359%	94%	988%	137%	4%	442%			
7	AAV01	43%	14%	78%	-38%	21%	58%	44%	14%	82%	42%	13%	78%	57%	14%	115%						
8	AAZ01	-27%	-51%	9%	-46%	-60%	-26%	-25%	-51%	14%	-25%	-50%	12%				-37%	-65%	12%			
9	ABA03	50%	14%	97%	-25%	3%	52%	54%	15%	105%	52%	15%	102%	64%	18%	129%	42%	-18%	144%			
10	AFA01	----			----			----			----			----			----					
11	AFA02	-6%	-16%	6%	-1%	-11%	10%	-8%	-19%	5%	-5%	-17%	9%	-10%	-27%	10%						
12	AHA01	13%	-2%	31%	-6%	-17%	6%	15%	-1%	34%	15%	-1%	34%	37%	-29%	166%	42%	-8%	119%			
Parity																						
	1	----			----			----			----			----			----					
	2	-36%	-40%	-33%	29%	-33%	-25%	-38%	-42%	-34%	-38%	-42%	-34%	-38%	-43%	-33%	-29%	-34%	-23%			
	3 or more	-47%	-51%	-44%	39%	-43%	-35%	-50%	-54%	-45%	-50%	-54%	-45%	-48%	-54%	-42%	-40%	-45%	-34%			
Trimester																						
	1	----			----			----			----			----			----					
	2	-7%	-20%	8%	7%	-18%	7%	-7%	-20%	9%	-10%	-24%	7%	5%	-11%	25%	-9%	-24%	8%			
	3	-15%	-28%	-1%	10%	-22%	4%	-16%	-28%	-1%	-17%	-30%	-2%	-3%	-21%	19%	-7%	-36%	35%			
BMI																						
	BMICAT1										----											
	BMICAT2										-3%	-19%	16%									
	BMICAT3										-1%	-18%	18%									
	BMICAT4										-3%	-20%	16%									
Breast feeding ever																						
0	no	----			----			----			----			----			----					
1	yes													27%	2%	59%						
Fish consumption																	----					
	0-0.23 per week																3%	-6%	14%			
	0.23-0.92 per week																3%	-7%	14%			
	0.92-1.69 per week																4%	-6%	15%			
	>1.69 per week																					
PFOS																						
	Quartile 1				----																	
	Quartile 2				72%	61%	84%															
	Quartile 3				131%	115%	148%															
	Quartile 4				205%	174%	239%															

Footnote: Some college, no degree; Associate's degree (AA, AS); Trade school; , GED or equivalent; (BA, BS) and above
