# Supplementary material for: Sociodemographic predictors of PFAS exposure among a combined sample of U.S. pregnant women: an Environmental influences on Child Health Outcomes (ECHO) public-use dataset analysis
Source: J Expo Sci Environ Epidemiol. 2025 Dec 15;36(3):459–68. doi: 10.1038/s41370-025-00833-8 (PMC13143815; doi:10.1038/s41370-025-00833-8)
Supplement: Supplementary file 5 — Supplementary Table5 [file 41370_2025_833_MOESM5_ESM.pdf]

Supplemental Table 5: PFOS, includes estimated percent difference adjusted for race, ethnicity, education, cohort, parity, trimester, maternal age, and year of sample collection and 95% interval for final model, model adjusted for quartile of PFOS, model with Cohort #6 restricted, model adjusted for BMI, breast feeding, and weekly fish consumption

Mean (SD)		PFOS n=15,215			quartiles of PFOA (sensitivity analysis) n=15,215			PFOS; no AAU01 cohort (sensitivity analysis) n=12,455			PFOS (including BMI) n=12,910			PFOS (including breastfeeding) n=7572			PFOS (including FISH) n=7,460			PFOS (unadjusted) n=15,125		
		%change	95 % CI		%change	95 % CI		%change	95 % CI		%change	95 % CI		%change	95 % CI		%change	95 % CI		%change	95 % CI	
Race																						
1	White	----			----			----			----			----			----			----		
2	Black	-9%	-16%	-1%	1%	-7%	8%	-13%	-21%	-5%	-13%	-21%	-4%	-13%	-27%	5%	-4%	-13%	6%	-22%	-30%	-13%
3	Asian	0%	-10%	11%	-1%	-10%	10%	-1%	-12%	12%	3%	-9%	16%	1%	-15%	21%	-3%	-16%	12%	-14%	-28%	4%
4	Other	-10%	-22%	3%	-7%	17%	5%	-12%	-25%	2%	-13%	-25%	0%	-15%	-29%	1%	-6%	-22%	14%	-23%	-39%	-2%
Ethnicity																						
0	Non-Hispanic	----			----			----			----			----			----			----		
1	Hispanic	-18%	-24%	-12%	-16%	-21%	-10%	-20%	-26%	-13%	-15%	-23%	-7%	-16%	-25%	-5%	-16%	-24%	-8%	-49%	-54%	-44%
Maternal education																						
1	Less than high school	----			----			----			----			----			----			----		
2	High school degree	5%	-6%	17%	0%	-9%	11%	5%	-7%	17%	2%	-11%	17%	6%	-11%	28%	-2%	-17%	15%	45%	20%	76%
3	Some college	17%	5%	31%	8%	-2%	18%	13%	1%	26%	16%	1%	32%	19%	1%	40%	13%	-3%	33%	104%	70%	145%
4	Bachelor's degree	17%	4%	30%	6%	-4%	16%	18%	6%	33%	13%	-2%	29%	18%	-1%	40%	5%	-11%	24%	155%	116%	201%
Cohort																						
1	AAA01	29%	7%	56%	26%	7%	49%	32%	9%	60%	26%	3%	53%	47%	19%	83%	59%	1%	151%			
2	AAF01	99%	54%	158%	37%	15%	64%	103%	59%	159%	96%	52%	152%	107%	40%	207%						
3	AAG01	10%	-6%	-29%	-6%	-19%	8%	16%	-1%	37%	12%	-7%	36%	49%	18%	87%	21%	-23%	90%			
4	AAP01	-10%	-22%	-4%	11%	-2%	25%	-8%	-20%	6%	-14%	-26%	-1%	6%	-17%	36%						
5	AAS01	96%	45%	165%	31%	7%	60%	108%	56%	177%				66%	-19%	242%	46%	-21%	170%			
6	AAU01	494%	189%	1123%	252%	122%	457%				501%	192%	1138%	530%	109%	1800%	114%	-1%	362%			
7	AAV01	11%	-10%	-38%	-12%	-24%	2%	14%	-7%	40%	10%	-12%	36%	20%	-17%	73%						
8	AAZ01	94%	29%	192%	81%	34%	144%	106%	39%	206%	100%	32%	203%				41%	-21%	150%			
9	ABA03	55%	21%	100%	23%	2%	49%	61%	26%	105%	53%	19%	98%	66%	17%	136%	43%	-14%	139%			
10	AFA01	----			----			----			----			----			----	----	----			
11	AFA02	-10%	-19%	-1%	-8%	-17%	1%	-10%	-20%	1%	-9%	-20%	3%	-5%	-24%	19%						
12	AHA01	52%	32%	76%	51%	34%	70%	52%	32%	75%	51%	31%	75%	80%	28%	151%	134%	53%	257%			
Parity																						
	1	----			----			----			----			----								
	2	-21%	-26%	-17%	-5%	-10%	0%	-22%	-27%	-17%	-23%	-27%	-18%	-23%	-29%	-16%						
	3 or more	-30%	-34%	-25%	-9%	-15%	-3%	-31%	-36%	-26%	-32%	-37%	-27%	-31%	-37%	-24%						
Trimester																						
	1	----			----			----			----			----			----					
	2	-6%	-17%	6%	-4%	-14%	7%	-7%	-17%	5%	-7%	-18%	6%	-1%	-14%	15%	1%	-16%	23%			
	3	-17%	-27%	-6%	-12%	-21%	-1%	-17%	-28%	-6%	-17%	-28%	-4%	-8%	-23%	10%	2%	-31%	53%			
BMI																						
	BMICAT1										----											
	BMICAT2										4%	-12%	23%									
	BMICAT3										6%	-11%	26%									
	BMICAT4										0%	-16%	20%									
Breast feeding ever																						
0	no	----			----			----			----			----			----					
1	yes																					
Fish consumption																	----					
	0-0.23 per week													----			1%	-8%	11%			
	0.23-0.92 per week																1%	-8%	12%			
	0.92-1.69 per week																2%	-8%	12%			
	>1.69 per week																					
PFOA																						
	Quartile 1				----																	
	Quartile 2				67%	56%	78%															
	Quartile 3				140%	122%	158%															
	Quartile 4				240%	207%	277%															

Footnote: Some college, no degree; Associate's degree (AA, AS); Trade school; , GED or equivalent; (BA, BS) and above
