# Supplementary material for: Sociodemographic predictors of PFAS exposure among a combined sample of U.S. pregnant women: an Environmental influences on Child Health Outcomes (ECHO) public-use dataset analysis
Source: J Expo Sci Environ Epidemiol. 2025 Dec 15;36(3):459–68. doi: 10.1038/s41370-025-00833-8 (PMC13143815; doi:10.1038/s41370-025-00833-8)
Supplement: Supplementary file 6 — Supplementary Table6 [file 41370_2025_833_MOESM6_ESM.pdf]

Supplemental Table 6: PFHxS, includes estimated percent difference adjusted for race, ethnicity, education, cohort, parity, trimester, maternal age, and year of sample collection and 95% interval for final model, model with Cohort #6 restricted, model adjusted for BMI, breast feeding, and weekly fish consumption

		PFHxS n=15,215			PFHxS; no AAU01 cohort (sensitivity analysis) n=12,455			PFHXS (including BMI) n=12,910			PFHXS (including breastfeeding) n=7,572			PFHXS (including FISH) n=6,870			PFHXS (unadjusted)n=15,125		
		%change	95 % CI		%change	95 % CI		%change	95 % CI		%change	95 % CI		%change	95 % CI		%change	95 % CI	
Race																			
1	White	----			----			----			----			----			----		
2	Black	-23%	-30%	-16%	-22%	-30%	-13%	-24%	-32%	-15%	-22%	-34%	-8%	-19%	-28%	-8%	-7%	-16%	2%
3	Asian	-25%	-33%	-16%	-26%	-35%	-16%	-24%	-33%	-14%	-23%	-40%	-1%	-24%	-37%	-8%	-34%	-43%	-23%
4	Other	-19%	-31%	-6%	-21%	-33%	-7%	-19%	-31%	-5%	-23%	-38%	-6%	-10%	-29%	14%	-26%	-39%	-10%
Ethnicity																			
0	Non-Hispanic	----			----			----			----			----			----		
1	Hispanic	-29%	-35%	-23%	-30%	-36%	-24%	-27%	-34%	-19%	-24%	-34%	-12%	-19%	-29%	-8%	-30%	-36%	-23%
Maternal education																			
1	Less than high school	----			----			----			----			----			----		
2	High school degree	6%	-6%	21%	4%	-8%	18%	1%	-13%	17%	7%	-12%	29%	14%	-7%	39%	29%	10%	53%
3	Some college	17%	3%	32%	15%	1%	31%	8%	-7%	25%	14%	-6%	38%	23%	1%	52%	46%	25%	71%
4	Bachelor's degree	12%	-1%	27%	14%	0%	29%	1%	-12%	17%	4%	-15%	26%	16%	-6%	44%	49%	30%	72%
Cohort																			
1	AAA01	217%	154%	294%	223%	160%	301%	207%	146%	283%	203%	134%	293%	189%	69%	395%			
2	AAF01	1%	-21%	30%	4%	-18%	33%	2%	-21%	31%	-3%	-27%	30%						
3	AAG01	169%	122%	225%	170%	123%	226%	168%	117%	231%	177%	114%	260%	119%	28%	277%			
4	AAP01	226%	176%	285%	232%	182%	292%	207%	157%	266%	205%	139%	288%						
5	AAS01	37%	2%	84%	43%	8%	91%				18%	-18%	68%	19%	-44%	154%			
6	AAU01	98%	3%	281%				103%	4%	298%	88%	-20%	345%	47%	-45%	295%			
7	AAV01	17%	-6%	45%	20%	-3%	48%	16%	-7%	43%	10%	-21%	52%						
8	AAZ01	-8%	-36%	31%	-4%	-32%	36%	-9%	-37%	30%				-20%	-60%	62%			
9	ABA03	-5%	-27%	23%	0%	-23%	29%	-8%	-29%	20%	-10%	-33%	22%	-16%	-55%	56%			
10	AFA01	----			----			----			----			----					
11	AFA02	-30%	-38%	-20%	-30%	-39%	-21%	-25%	-35%	-14%	-38%	-50%	-23%						
12	AHA01	335%	269%	411%	333%	269%	408%	327%	263%	402%	353%	178%	639%	337%	170%	607%			
Parity																			
	1	----			----			----			----			----					
	2	-24%	-29%	-19%	-26%	-31%	-20%	-24%	-29%	-18%	-23%	-30%	-16%	-15%	-23%	-6%			
	3 or more	-37%	-42%	-32%	-39%	-44%	-34%	-38%	-43%	-32%	-39%	-46%	-30%	-29%	-37%	-21%			
Trimester																			
	1	----			----			----			----			----					
	2	11%	-3%	27%	10%	-4%	26%	-1%	-19%	21%	12%	-7%	34%	-2%	-22%	23%			
	3	21%	4%	42%	21%	4%	40%	-10%	-26%	10%	27%	4%	55%	11%	-31%	78%			
BMI																			
	BMICAT1							----											
	BMICAT2							2%	-15%	24%									
	BMICAT3							3%	-15%	25%									
	BMICAT4							-1%	-19%	21%									
Breast feeding ever																			
0	no	----						----			----			----					
1	yes										36%	6%	74%	----					
Fish consumption														----					
	0-0.23 per week										----			7%	-6%	20%			
	0.23-0.92 per week													9%	-4%	24%			
	0.92-1.69 per week													7%	-5%	22%			
	>1.69 per week																		
Breastfeeding																			
PFOS																			
	Quartile 1																		
	Quartile 2																		
	Quartile 3																		
	Quartile 4																		

Footnote: Some college, no degree; Associate's degree (AA, AS); Trade school; , GED or equivalent; (BA, BS) and above
