# Supplementary material for: Sociodemographic predictors of PFAS exposure among a combined sample of U.S. pregnant women: an Environmental influences on Child Health Outcomes (ECHO) public-use dataset analysis
Source: J Expo Sci Environ Epidemiol. 2025 Dec 15;36(3):459–68. doi: 10.1038/s41370-025-00833-8 (PMC13143815; doi:10.1038/s41370-025-00833-8)
Supplement: Supplementary file 7 — Supplementary Table7 [file 41370_2025_833_MOESM7_ESM.pdf]

Supplemental Table 7: PFNA, includes estimated percent difference adjusted for race, ethnicity, education, cohort, parity, trimester, maternal age, and year of sample collection and 95% interval for final model, model with Cohort #6 restricted, model adjusted for BMI, breast feeding, and weekly fish consumption

		PFNA n=15,210			PFNA; no AAU01 cohort (sensitivity analysis) n=12,450			PFNA (including BMI) n=13,065			PFNA (including breastfeeding) n=7,572			PFNA (including FISH) n=6,870			PFNA (unadjusted) n=15,120		
		%change	95 % CI		%change	95 % CI		%change	95 % CI		%change	95 % CI		%change	95 % CI		%change	95 % CI	
Race																			
1	White	----			----			----			----			----			----		
2	Black	-5%	-12%	3%	5%	14%	4%	10%	19%	0%	8%	21%	6%	7%	4%	19%	13%	20%	6%
3	Asian	26%	13%	40%	23%	10%	38%	28%	14%	45%	32%	13%	54%	31%	13%	52%	22%	7%	40%
4	Other	-3%	-16%	11%	3%	17%	14%	2%	16%	13%	10%	25%	10%	16%	31%	3%	11%	26%	7%
Ethnicity																			
0	Non-Hispanic	----			----			----			----			----			----		
1	Hispanic	-3%	-10%	5%	4%	12%	4%	3%	12%	6%	0%	15%	17%	8%	3%	19%	43%	47%	38%
Maternal education																			
1	Less than high school	----			----			----			----			----			----		
2	High school degree	8%	-4%	21%	8%	4%	22%	7%	7%	23%	7%	10%	28%	1%	16%	17%	47%	27%	69%
3	Some college	20%	7%	35%	20%	6%	35%	19%	4%	37%	20%	3%	48%	9%	8%	29%	88%	64%	116%
4	Bachelor's degree	24%	11%	39%	26%	12%	42%	25%	9%	44%	28%	1%	62%	4%	13%	25%	122%	95%	152%
Cohort																			
1	AAA01	28%	5%	56%	30%	7%	59%	29%	6%	58%	38%	9%	74%	28%	20%	105%			
2	AAF01	102%	40%	191%	103%	39%	196%	104%	43%	191%	119%	46%	230%						
3	AAG01	13%	-5%	33%	13%	5%	35%	22%	1%	49%	36%	6%	75%	6%	33%	68%			
4	AAP01	-55%	-62%	-47%	54%	61%	46%	54%	62%	45%	52%	67%	29%						
5	AAS01	59%	4%	142%	61%	4%	150%				46%	22%	172%	23%	36%	136%			
6	AAU01	-20%	-71%	125%				14%	69%	136%	4%	66%	221%	66%	85%	22%			
7	AAV01	30%	-4%	76%	31%	4%	80%	32%	2%	77%	43%	1%	108%						
8	AAZ01	60%	-7%	176%	61%	8%	182%	70%	0%	190%				12%	39%	105%			
9	ABA03	71%	22%	141%	74%	22%	148%	77%	26%	148%	86%	24%	178%	44%	16%	146%			
10	AFA01	----			----			----			----			----					
11	AFA02	-2%	-13%	10%	2%	13%	11%	0%	11%	14%	4%	21%	16%						
12	AHA01	25%	6%	48%	25%	5%	49%	27%	7%	51%	54%	13%	171%	54%	2%	135%			
Parity																			
	1	----			----			----			----			----					
	2	-24%	-28%	-19%	24%	29%	18%	25%	30%	20%	24%	30%	17%	20%	26%	14%			
	3 or more	-31%	-36%	-26%	30%	35%	24%	33%	38%	28%	30%	37%	22%	27%	34%	20%			
Trimester																			
	1	----			----			----			----			----					
	2	-5%	-16%	8%	5%	16%	8%	4%	17%	10%	3%	17%	13%	13%	27%	5%			
	3	-14%	-26%	1%	14%	26%	1%	12%	26%	4%	13%	28%	6%	20%	46%	19%			
BMI																			
	BMICAT1							----											
	BMICAT2							6%	21%	12%									
	BMICAT3							6%	21%	12%									
	BMICAT4							5%	21%	13%									
Breast feeding ever																			
0	no	----			----			----			----			----					
1	yes										18%	5%	47%						
Fish consumption														----					
	0-0.23 per week										----			5%	5%	15%			
	0.23-0.92 per week													13%	2%	25%			
	0.92-1.69 per week													16%	5%	29%			
	>1.69 per week																		
PFOS																			
	Quartile 1																		
	Quartile 2																		
	Quartile 3																		
	Quartile 4																		

Footnote: Some college, no degree; Associate's degree (AA, AS); Trade school; , GED or equivalent; (BA, BS) and above
