# Supplementary material for: Sociodemographic predictors of PFAS exposure among a combined sample of U.S. pregnant women: an Environmental influences on Child Health Outcomes (ECHO) public-use dataset analysis
Source: J Expo Sci Environ Epidemiol. 2025 Dec 15;36(3):459–68. doi: 10.1038/s41370-025-00833-8 (PMC13143815; doi:10.1038/s41370-025-00833-8)
Supplement: Supplementary file 8 — Supplementary Table8 [file 41370_2025_833_MOESM8_ESM.pdf]

Supplemental Table 8: Sum of four PFAS (PFOA, PFOS, PFHxS, PFNA) includes estimated percent difference adjusted for race, ethnicity, education, cohort, parity, trimester, maternal age, and year of sample collection and 95% interval for final model, model with Cohort #6 restricted, model adjusted for BMI, breast feeding, and weekly fish consumption

		Sum PFAS n=15,215			Sum PFAS; no AAU01 cohort (sensitivity analysis) n=12,455			Sum PFAS (including BMI) n=12,910			Sum PFAS (including breastfeeding) n=7,572			Sum PFAS (including FISH) n=7,460			Sum PFAS (unadjusted) n=15,125		
		%change	95 % CI		%change	95 % CI		%change	95 % CI		%change	95 % CI		%change	95 % CI		%change	95 % CI	
Race																			
1	White	----			----			----			----			----			----		
2	Black	-11%	-16%	-4%	-13%	-20%	-6%	-12%	-19%	-5%	-15%	-25%	-4%	-6%	-14%	1%	-20%	-27%	-12%
3	Asian	-3%	-11%	6%	-3%	-11%	7%	0%	-9%	10%	1%	-13%	18%	-6%	-16%	6%	-15%	-27%	-1%
4	Other	-9%	-18%	2%	-10%	-20%	2%	-9%	-19%	2%	-14%	-26%	0%	-10%	-23%	4%	-20%	-34%	-3%
Ethnicity																			
0	Non-Hispanic	----			----			----			----			----			----		
1	Hispanic	-16%	-21%	-11%	-17%	-22%	-11%	-15%	-21%	-8%	-14%	-22%	-5%	-11%	-18%	-4%	-45%	-49%	-39%
Maternal education																			
1	Less than high school	----			----			----			----			----			----		
2	High school degree	7%	-2%	18%	7%	-3%	18%	5%	-7%	18%	8%	-7%	25%	4%	-9%	20%	44%	22%	70%
3	Some college	19%	8%	30%	15%	4%	26%	15%	3%	29%	18%	2%	37%	20%	4%	38%	92%	64%	124%
4	Bachelor's degree	17%	5%	29%	18%	6%	31%	12%	-1%	27%	16%	-1%	36%	10%	-5%	28%	131%	100%	166%
Cohort																			
1	AAA01	42%	21%	66%	44%	23%	70%	40%	19%	64%	54%	27%	87%	61%	15%	126%			
2	AAF01	61%	20%	115%	64%	23%	118%	59%	19%	113%	70%	24%	134%						
3	AAG01	36%	19%	55%	42%	24%	63%	38%	19%	61%	68%	38%	104%	35%	-4%	89%			
4	AAP01	8%	-5%	21%	10%	-3%	24%	4%	-8%	19%	17%	-1%	40%						
5	AAS01	60%	14%	126%	69%	20%	137%				51%	-18%	178%	27%	-21%	105%			
6	AAU01	273%	57%	787%				281%	58%	821%	317%	69%	930%	94%	4%	261%			
7	AAV01	13%	-12%	45%	16%	-10%	48%	11%	-13%	44%	21%	-10%	63%						
8	AAZ01	54%	1%	137%	63%	7%	149%	57%	2%	141%				28%	-18%	100%			
9	ABA03	34%	2%	76%	39%	6%	82%	34%	1%	76%	43%	5%	96%	24%	-16%	83%			
10	AFA01	----			----			----			----			----					
11	AFA02	-12%	-20%	-3%	-13%	-21%	-4%	-11%	-20%	-1%	-13%	-26%	2%						
12	AHA01	81%	58%	106%	81%	58%	107%	80%	57%	107%	114%	38%	230%	136%	75%	220%			
Parity																			
	1	----			----			----			----			----					
	2	-25%	-28%	-21%	-26%	-29%	-22%	-26%	-29%	-22%	-25%	-30%	-19%	-18%	-22%	-12%			
	3 or more	-34%	-38%	-30%	-35%	-39%	-31%	-36%	-40%	-32%	-35%	-40%	-29%	-27%	-32%	-22%			
Trimester																			
	1	----			----			----			----			----					
	2	2%	-8%	12%	1%	-8%	12%	2%	-9%	13%	4%	-9%	18%	-10%	-21%	3%			
	3	-3%	-13%	8%	-3%	-13%	8%	-2%	-12%	10%	0%	-14%	17%	-2%	-27%	32%			
BMI																			
	BMICAT1							----											
	BMICAT2							0%	-13%	14%									
	BMICAT3							1%	-12%	17%									
	BMICAT4							-3%	-15%	12%									
Breast feeding ever																			
0	no	----			----														
1	yes																		
Fish consumption														----					
	0-0.23 per week													1%	-8%	11%			
	0.23-0.92 per week													1%	-8%	12%			
	0.92-1.69 per week													2%	-8%	12%			
	>1.69 per week																		
PFOS																			
	Quartile 1																		
	Quartile 2																		
	Quartile 3																		
	Quartile 4																		

Footnote: Some college, no degree; Associate's degree (AA, AS); Trade school; , GED or equivalent; (BA, BS) and above
