# Supplementary material for: Sociodemographic predictors of PFAS exposure among a combined sample of U.S. pregnant women: an Environmental influences on Child Health Outcomes (ECHO) public-use dataset analysis
Source: J Expo Sci Environ Epidemiol. 2025 Dec 15;36(3):459–68. doi: 10.1038/s41370-025-00833-8 (PMC13143815; doi:10.1038/s41370-025-00833-8)
Supplement: Supplementary file 9 — Supplementary Table9 [file 41370_2025_833_MOESM9_ESM.pdf]

Supplemental Table 9: NMFOSAA includes estimated percent difference adjusted for race, ethnicity, education, cohort, parity, trimester, maternal age, and year of sample collection and 95% interval for final model, model with Cohort #6 restricted, model adjusted for BMI, breast feeding, and weekly fish consumption

		NMFOSAA n=13,130			NMFOSAA; no AAU01 cohort (sensitivity analysis) n=13,390			NMFOSAA (including BMI) n=12,485			NMFOSAA (including breastfeeding) n=7,047			NMFOSAA (including FISH) n=7,460			NMFOSAA (unadjusted) n=15,125		
		%change	95 % CI		%change	95 % CI		%change	95 % CI		%change	95 % CI		%change	95 % CI		%change	95 % CI	
Race																			
1	White	----			----			----			----			----			----		
2	Black	1%	-13%	17%	0%	-17%	21%	3%	-12%	20%	4%	-19%	33%	3%	-18%	29%	-11%	-27%	9%
3	Asian	-15%	-29%	1%	-15%	-32%	5%	-16%	-30%	1%	-8%	-30%	20%	-9%	-32%	22%	-37%	-53%	-15%
4	Other	6%	-17%	35%	10%	-18%	47%	5%	-18%	35%	19%	-21%	79%	-7%	-36%	34%	-10%	-39%	31%
Ethnicity																			
0	Non-Hispanic	----			----			----			----			----			----		
1	Hispanic	-14%	-24%	-3%	-15%	-26%	-1%	-14%	-24%	1%	-22%	-39%	-1%	-18%	-34%	2%	-50%	-58%	-41%
Maternal education																			
1	Less than high school	----			----			----			----			----			----		
2	High school degree	-3%	-24%	23%	-4%	-25%	23%	-8%	-26%	15%	-8%	-35%	29%	-18%	-46%	25%	35%	-7%	96%
3	Some college	-5%	-24%	18%	-9%	-27%	14%	-8%	-26%	14%	-13%	-37%	20%	-18%	-46%	25%	102%	45%	182%
4	Bachelor's degree	-9%	-30%	19%	-11%	-32%	17%	-14%	-34%	12%	-18%	-42%	15%	-21%	-48%	19%	134%	68%	226%
Cohort																			
1	AAA01	-30%	-50%	-2%	-31%	-52%	-1%	-31%	-50%	4%	-32%	-53%	-1%	-50%	-74%	-3%			
2	AAF01	20%	-7%	54%	25%	-4%	63%	19%	-9%	54%	21%	-16%	74%						
3	AAG01	-23%	-41%	0%	-23%	-42%	3%	-24%	-43%	0%	-28%	-52%	9%	-50%	-74%	-1%			
4	AAP01	-20%	-38%	2%	-21%	-39%	3%	-22%	-39%	1%	-22%	-47%	13%						
5	AAS01				0%	0%	0%												
6	AAU01	1854%	1069%	3166%				1945%	1107%	3367%	2078%	798%	5186%	272%	15%	1104%			
7	AAV01	-7%	-26%	17%	-4%	-24%	22%	-7%	-27%	19%	1%	-48%	94%						
8	AAZ01				0%	0%	0%	0%	0%	0%									
9	ABA03	23%	-10%	68%	30%	-6%	80%	20%	-14%	67%	22%	-22%	89%	-42%	-72%	19%			
10	AFA01	----			----			----			----			----					
11	AFA02	-10%	-24%	8%	-13%	-28%	4%	-6%	-22%	13%	-26%	-47%	3%						
12	AHA01	-17%	-37%	8%	-19%	-38%	6%	-19%	-38%	6%	-16%	-53%	51%	-32%	-63%	26%			
Parity																			
	1	----			----			----			----			----					
	2	0%	-11%	12%	-6%	-19%	9%	0%	-11%	13%	-5%	-23%	17%	11%	-2%	26%			
	3 or more	3%	-8%	16%	-2%	-15%	13%	5%	-7%	18%	2%	-15%	23%	16%	-2%	36%			
Trimester																			
	1	----			----			----			----			----					
	2	2%	-20%	30%	2%	-21%	31%	3%	-19%	31%	3%	-30%	52%	-3%	-36%	46%			
	3	-7%	-28%	22%	-8%	-30%	22%	-6%	-28%	23%	-6%	-42%	51%	19%	-34%	117%			
BMI																			
	BMICAT1							----											
	BMICAT2							9%	-10%	32%									
	BMICAT3							7%	-12%	30%									
	BMICAT4							5%	-14%	29%									
Breast feeding ever																			
0	no	----									----								
1	yes										-17%	-46%	26%						
Fish consumption														----					
	0-0.23 per week													-6%	-27%	22%			
	0.23-0.92 per week													-10%	-31%	17%			
	0.92-1.69 per week													-5%	-29%	26%			
	>1.69 per week																		
PFOS																			
	Quartile 1																		
	Quartile 2																		
	Quartile 3																		
	Quartile 4																		

Footnote: Some college, no degree; Associate's degree (AA, AS); Trade school; , GED or equivalent; (BA, BS) and above
