# Supplementary material for: Sociodemographic predictors of PFAS exposure among a combined sample of U.S. pregnant women: an Environmental influences on Child Health Outcomes (ECHO) public-use dataset analysis
Source: J Expo Sci Environ Epidemiol. 2025 Dec 15;36(3):459–68. doi: 10.1038/s41370-025-00833-8 (PMC13143815; doi:10.1038/s41370-025-00833-8)
Supplement: Supplementary file 10 — Supplementary Table10 [file 41370_2025_833_MOESM10_ESM.pdf]

Supplemental Table 10: PFDA includes estimated percent difference adjusted for race, ethnicity, education, cohort, parity, trimester, maternal age, and year of sample collection and 95% interval for final model, model with Cohort #6 restricted, model adjusted for BMI, breast feeding, and weekly fish consumption

			PFDA n=12,970			PFDA; no AAU01 cohort (sensitivity analysis) n=10,210			PFDA (including BMI) n=12,450			PFDA (including breastfeeding) n=7,077			PFDA (including FISH) n=4,795			PFDA (unadjusted) n=12,970		
			%change	95 % CI		%change	95 % CI		%change	95 % CI		%change	95 % CI		%change	95 % CI		%change	95 % CI	
Race																				
1	White		----			----			----			----			----			----		
2	Black		-12%	-25%	2%	-14%	-29%	4%	-10%	-23%	6%	-11%	-31%	13%	-7%	-28%	20%	-30%	-38%	-21%
3	Asian		60%	36%	89%	55%	31%	83%	62%	37%	92%	73%	32%	127%	76%	28%	141%	69%	43%	99%
4	Other		2%	-19%	29%	8%	-13%	33%	2%	-19%	27%	-10%	-32%	20%	-18%	-46%	26%	-3%	-23%	22%
Ethnicity																				
0	Non-Hispanic		----			----			----			----			----			----		
1	Hispanic		2%	-11%	17%	-1%	-14%	14%	6%	-8%	23%	11%	-7%	33%	8%	-18%	43%	-21%	-29%	-12%
Maternal education																				
1	Less than high school		----			----			----			----			----			----		
2	High school degree		-8%	-31%	22%	-1%	-25%	30%	-7%	-30%	22%	4%	-46%	102%	-17%	-55%	52%	-4%	-26%	24%
3	Some college		3%	-20%	32%	5%	-18%	33%	5%	-18%	33%	19%	-39%	132%	-10%	-54%	75%	17%	-8%	50%
4	Bachelor's degree		17%	-7%	48%	19%	-8%	53%	15%	-9%	46%	34%	-33%	170%	2%	-45%	89%	54%	26%	88%
Cohort																				
1	AAA01		-4%	-27%	27%	-3%	-25%	25%	-4%	-28%	28%	4%	-23%	40%	-27%	-69%	70%			
2	AAF01		75%	22%	153%	75%	20%	156%	76%	22%	152%	104%	39%	199%						
3	AAG01		18%	-10%	54%	12%	-15%	48%	25%	-8%	70%	41%	-4%	109%	-7%	-64%	141%			
4	AAP01		4%	-23%	40%	3%	-21%	35%	3%	-25%	43%	9%	-29%	69%						
5	AAS01		0%	0%	0%										0%	0%	0%			
6	AAU01		-39%	-78%	74%				-36%	-77%	81%	-36%	-80%	110%	-90%	-98%	-44%			
7	AAV01		30%	-1%	70%	28%	-3%	68%	28%	-2%	67%	50%	4%	119%						
8	AAZ01		0%	0%	0%				0%	0%	0%				0%	0%	0%			
9	ABA03		33%	-3%	83%	33%	-2%	78%	32%	-4%	80%	51%	0%	128%	-23%	-75%	133%			
10	AFA01		----			----			----			----			----					
11	AFA02		51%	26%	80%	54%	30%	83%	50%	24%	83%									
12	AHA01		-1%	-26%	32%	-5%	-27%	26%	-2%	-28%	33%	35%	-37%	190%	6%	-52%	136%			
Parity																				
		1	----			----			----			----			----					
		2	-20%	-27%	-12%	-17%	-25%	-8%	-19%	-27%	-11%	-19%	-30%	-6%	-23%	-36%	-6%			
	3 or more		-24%	-33%	-15%	-20%	-29%	-9%	-25%	-34%	-15%	-25%	-39%	-8%	-29%	-44%	-11%			
Trimester																				
		1	----			----			----			----			----					
		2	-2%	-21%	23%	-1%	-20%	22%	4%	-19%	33%	-4%	-26%	25%	-5%	-33%	35%			
		3	-9%	-30%	18%	-10%	-30%	16%	-6%	-28%	25%	-15%	-38%	15%	-1%	-55%	119%			
BMI																				
	BMICAT1								----											
	BMICAT2								3%	-28%	47%									
	BMICAT3								1%	-28%	44%									
	BMICAT4								-10%	-38%	30%									
Breast feeding ever																				
0	no		----			----			----			----			----					
1	yes											27%	-22%	106%						
Fish consumption															----					
	0-0.23 per week											----			8%	-17%	40%			
	0.23-0.92 per week														26%	-5%	66%			
	0.92-1.69 per week														47%	10%	95%			
	>1.69 per week																			
PFOS																				
	Quartile 1																			
	Quartile 2																			
	Quartile 3																			
	Quartile 4																			

Footnote: Some college, no degree; Associate's degree (AA, AS); Trade school; , GED or equivalent; (BA, BS) and above
