# Supplementary material for: Sociodemographic predictors of PFAS exposure among a combined sample of U.S. pregnant women: an Environmental influences on Child Health Outcomes (ECHO) public-use dataset analysis
Source: J Expo Sci Environ Epidemiol. 2025 Dec 15;36(3):459–68. doi: 10.1038/s41370-025-00833-8 (PMC13143815; doi:10.1038/s41370-025-00833-8)
Supplement: Supplementary file 11 — Supplementary Table11 [file 41370_2025_833_MOESM11_ESM.pdf]

Supplemental Table 11: PFUnDA includes estimated percent difference adjusted for race, ethnicity, education, cohort, parity, trimester, maternal age, and year of sample collection and 95% interval for final model, model with Cohort #6 restricted, model adjusted for BMI, breast feeding, and weekly fish consumption																				
			PFUNDA n=8,620			PFUNDA; not run because AAU01 did not measure PFUNDA and is not included cohort			PFUNDA (including BMI) n=8,150			PFUNDA (including breastfeeding) n=4966			PFUNDA (including FISH) n=2,285			PFUNDA (unadjusted) n=8,620		
			%change	95 % CI		%change	95 % CI		%change	95 % CI		%change	95 % CI		%change	95 % CI		%change	95 % CI	
Race																				
1	White		----			----			----			----			----			----		
2	Black		-1%	-22%	26%	0	0	0	3%	-20%	33%	2%	-30%	47%	-3%	-34%	41%	-15%	-29%	3%
3	Asian		95%	63%	134%	0	0	0	96%	62%	138%	120%	64%	195%	79%	22%	165%	171%	127%	224%
4	Other		0%	-24%	30%	0	0	0	-1%	-25%	30%	-11%	-37%	28%	-22%	-56%	38%	10%	-17%	45%
Ethnicity																				
0	Non-Hispanic		----			----			----			----			----			----		
1	Hispanic		-14%	-29%	5%	0	0	0	-10%	-26%	10%	-2%	-28%	32%	-3%	-38%	51%	-31%	-40%	-20%
Maternal education																				
1	Less than high school		----			----			----			----			----			----		
2	High school degree		-4%	-32%	36%	0	0	0	-4%	-32%	37%	-8%	-37%	33%	10%	-33%	79%	10%	-22%	54%
3	Some college		6%	-25%	50%	0	0	0	6%	-25%	50%	5%	32%	62%	8%	-40%	96%	25%	-12%	76%
4	Bachelor's degree		29%	-4%	73%	0	0	0	26%	-6%	71%	20%	22%	84%	29%	-29%	136%	79%	34%	139%
Cohort																				
1	AAA01		68%	26%	123%	0	0	0	66%	25%	120%	78%	19%	166%	49%	-42%	287%			
2	AAF01		16%	-13%	56%	0	0	0	17%	-14%	58%	43%	-3%	109%						
3	AAG01		13%	-25%	70%	0	0	0	13%	-27%	73%	21%	-28%	105%	6%	-57%	159%			
4	AAP01		-4%	-26%	25%	0	0	0	-6%	-29%	23%	3%	-37%	69%						
5	AAS01		0%	0%	0%	0	0	0												
6	AAU01		0%	0%	0%															
7	AAV01		0%	0%	0%	0	0	0												
8	AAZ01		15%	-32%	96%	0	0	0	14%	-34%	95%				5%	-73%	308%			
9	ABA03		21%	-17%	76%	0	0	0	20%	-18%	75%	36%	-17%	122%	19%	-59%	245%			
10	AFA01		----			----			----			----			----					
11	AFA02		87%	57%	123%	0	0	0	91%	58%	132%	124%	62%	210%						
12	AHA01		6%	-18%	35%	0	0	0	4%	-20%	35%	34%	-37%	187%	-4%	-56%	110%			
Parity																				
	1		----			----			----			----			----					
	2		-8%	-19%	4%	0	0	0	-8%	-19%	4%	-5%	-21%	13%	-10%	-28%	11%			
	3 or more		-11%	-25%	4%				-11%	-25%	6%	-11%	-29%	11%	-15%	-37%	14%			
Trimester																				
	1		----			----			----			----			----					
	2		2%	-23%	35%	0	0	0	5%	-21%	39%	0%	-28%	38%	9%	-20%	49%			
	3		5%	-18%	35%	0	0	0	7%	-17%	38%	-5%	-32%	33%	16%	-36%	111%			
BMI																				
	BMICAT1								----											
	BMICAT2								-8%	-35%	31%									
	BMICAT3								-8%	-35%	30%									
	BMICAT4								-17%	-40%	15%									
Breast feeding ever																				
0	no		----			----			----			----			----					
1	yes											17%	-29%	94%						
Fish consumption															----					
	0-0.23 per week											----			24%	-4%	61%			
	0.23-0.92 per week														21%	-9%	60%			
	0.92-1.69 per week														54%	12%	110%			
	>1.69 per week																			
PFOS																				
	Quartile 1																			
	Quartile 2																			
	Quartile 3																			
	Quartile 4																			
Footnote: Some college, no degree; Associate's degree (AA, AS); Trade school; , GED or equivalent; (BA, BS) and above																				
