# Supplementary material for: Sociodemographic predictors of PFAS exposure among a combined sample of U.S. pregnant women: an Environmental influences on Child Health Outcomes (ECHO) public-use dataset analysis
Source: J Expo Sci Environ Epidemiol. 2025 Dec 15;36(3):459–68. doi: 10.1038/s41370-025-00833-8 (PMC13143815; doi:10.1038/s41370-025-00833-8)
Supplement: Supplementary file 12 — Supplementary Table12 [file 41370_2025_833_MOESM12_ESM.pdf]

Supplemental Table 12. Count and percent of binary PFOSA, EtFOSAA, PFHpA, PFDoDA, PFBS, PFHxA, and PFPeA (<LOD, ≥LOD) by race, ethnicity, and maternal education

	**Race**					**Ethnicity**			**Maternal education**			
	White	Black	Asian	Other	p-value	Non-Hispanic	Hispanic	p-value	High school or less	Some college	College	p-value
**PFOSA**					***0.006***			***0.0007***				***<.0001***
<LOD	1412 (93%)	356(98%)	127 (95%)	79 (96%)		1602 (93%)	381 (98%)		387 (99%)	359 (95%)	1237 (92%)	
≥LOD	108 (7%)	9 (2%)	6 (5%)	3 (4%)		117 (7%)	9 (2%)		3 (1%)	20 (5%)	103 (8%)	
**EtFOSAA**					***0.0001***			***<.0001***				***<.0001***
<LOD	903 (64%)	261 (75%)	114 (76%)	60 (73%)		1141 (66%)	197(80%)		239 (84%)	251 (71%)	848 (63%)	
≥LOD	498 (35%)	85 (25%)	36 (24%)	22 (27%)		591 (34%)	50 (20%)		44 (16%)	103 (29%)	494 (37%)	
**PFHpA**					*0.2968*			***<.0001***				***0.0004***
<LOD	741 (87%)	236 (88%)	89 (83%)	48 (81%)		829 (84%)	285 (93%)		296 (91%)	204 (90%)	614 (83%)	
≥LOD	115 (13%)	31 (12%)	18 (17%)	11 (19%)		155 (16%)	20 (7%)		30 (9%)	22 (10%)	123 (17%)	
**PFDoDA**					***0.0023***			***0.0059***				*0.3887*
<LOD	649 (92%)	222 (97%)	97 (85%)	53 (93%)		903 (93%)	118 (87%)		169 (92%)	171 (90%)	681 (93%)	
≥LOD	53 (8%)	8 (3%)	17 (15%)	4 (7%)		64 (7%)	18 (13%)		15 (8%)	18 (10%)	49 (7%)	
**PFBS**					***<.0001***			***0.0253***				***0.0187***
<LOD	770 (96%)	232 (88%)	78 (96%)	50 (96%)		861 (94%)	269 (97%)		282 (91%)	205 (95%)	643 (96%)	
≥LOD	30 (4%)	32 (12%)	3 (4%)	2 (4%)		59 (6%)	8 (3%)		27 (9%)	11 (5%)	29 (4%)	
**PFHxA**					***<.0001***			***0.0036***				***<.0001***
<LOD	165 (96%)	133 (62%)	32 (97%)	21 (100%)		305 (78%)	46 (94%)		106 (68%)	70 (77%)	175 (91%)	
≥LOD	6 (4%)	81 (38%)	1 (3%)	0 (0%)		85 (22%)	3 (6%)		50 (32%)	21 (23%)	17 (9%)	
**PFPeA**					***<.0001***			***<.0001***				0.2058
<LOD	57 (33%)	117 (55%)	2 (6%)	6 (29%)		175 (45%)	7 (14%)		73 (48%)	34 (37%)	75 (39%)	
≥LOD	114 (67%)	97 (45%)	31 (94%)	15 (71%)		215 (55%)	42 (86%)		83 (53%)	57 (63%)	117 (61%)	
