# Supplementary material for: Sociodemographic predictors of PFAS exposure among a combined sample of U.S. pregnant women: an Environmental influences on Child Health Outcomes (ECHO) public-use dataset analysis
Source: J Expo Sci Environ Epidemiol. 2025 Dec 15;36(3):459–68. doi: 10.1038/s41370-025-00833-8 (PMC13143815; doi:10.1038/s41370-025-00833-8)
Supplement: Supplementary file 13 — Supplementary Table13 [file 41370_2025_833_MOESM13_ESM.pdf]

Supplemental 13. Associations of PFAS serum level ≥ LOD and sociodemographic factors unadjusted (crude) with increasing levels of adjustment

		**Race**			**Ethnicity**	**Maternal education**	
		White			non-Hispanic	High school or less	
		Reference			*Reference*	*Reference*	
	OR (95% CI)	Black	Asian	Other	Hispanic	Some college	College
**PFOSA**	Crude	**0.33 (0.17-0.66)**	0.62 (0.27-1.44)	0.50 (0.15-1.61)	0.32 (0.16-0.64)	**7.19 (2.12-24.39)**	**10.74 (3.39-34.04)**
	Adjusted 1	**0.43 (0.21-0.88)**	0.69 (0.29-1.62)	0.57 (0.18-1.87)	0.51 (0.25-1.04)	**5.39 (1.56-18.63)**	**7.52 (2.25-25.20)**
	Adjusted 2	0.50 (0.24-1.05)	0.64(0.27-1.51)	0.63 (0.19-2.06)	0.62 (0.29-1.30)	**4.82 (1.38-16.77)**	**5.72 (1.67-19.67)**
	Adjusted 3	0.67 (0.26-1.78)	0.32 (0.04-2.48)	0.34 (0.04-2.81)	0.99 (0.35-2.81)	7.71 (0.91-65.15)	4.88 (0.58-41.32)
**EtFOSAA**	Crude	**0.59 (0.45-0.77)**	**0.57 (0.39-0.85)**	0.66 (0.40-1.10)	**0.49 (0.36-0.68)**	**2.35 (1.57-3.52)**	**3.36 (2.37-4.77)**
	Adjusted 1	**0.53 (0.28-0.97)**	0.96 (0.48-1.92)	1.11 (0.43-2.88)	**0.51 (0.26-0.97)**	1.02 (0.48-2.15)	1.60 (0.82-3.12)
	Adjusted 2	**0.52 (0.27-0.99)**	0.92 (0.46-1.84)	1.29 (0.48-3.42)	0.50 (0.25-1.00)	0.91 (0.42-1.93)	1.19 (0.59-2.41)
	Adjusted 3	1.28 (0.29-5.65)	0.27 (0.04-1.78)	2.86 (0.28-29.36)	0.25 (0.06-1.01)	0.24 (0.05-1.15)	0.32 (0.08-1.31)
**PFHpA**	Crude	0.85 (0.55-1.29)	1.30 (0.76-2.24)	1.48 (0.74-2.93)	**0.38 (0.23-0.61)**	1.06 (0.60-1.90)	**1.98 (1.30-3.02)**
	Adjusted 1	1.12 (0.69-1.83)	1.11 (0.63-1.95)	1.57 (0.78-3.15)	**0.38 (0.22-0.66)**	1.09 (0.60-1.99)	1.58 (0.94-2.65)
	Adjusted 2	0.88 (0.50-1.56)	0.99 (0.56-1.74)	1.69 (0.83-3.45)	**0.38 (0.20-0.69)**	0.94 (0.50-1.76)	1.15 (0.64-2.05)
	Adjusted 3	1.10 (0.31-3.85)	1.51 (0.28-8.21)	3.72 (0.60-23.18)	0.60 (0.11-3.21)	1.13 (0.35-3.61)	1.02 (0.31-3.31)
**PFDoDA**	Crude	**0.44 (0.21-0.94)**	**2.15 (1.19-3.86)**	0.93 (0.32-2.65)	**2.15 (1.23-3.76)**	1.19 (0.58-2.43)	0.81 (0.44-1.48)
	Adjusted 1	**0.36 (0.14-0.93)**	1.84 (0.97-3.51)	0.74 (0.25-2.23)	1.66 (0.90-3.05)	1.08 (0.49-2.40)	0.75 (0.36-1.54)
	Adjusted 2	**0.32 (0.12-0.86)**	**1.98 (1.04-3.78)**	0.64 (0.21-1.96)	1.40 (0.71-2.76)	0.98 (0.43-2.25)	0.61 (0.27-1.37)
	Adjusted 3	0.40 (0.14-1.21)	**2.06 (0.85-5.00)**	-----	1.28 (0.52-3.16)	1.56 (0.54-4.49)	1.05 (0.38-2.90)
**PFBS**	Crude	**3.54 (2.11-5.95)**	0.99 (0.29-3.31)	1.03 (0.24-4.42)	**0.43 (0.20-0.92)**	0.56 (0.27-1.16)	**0.47 (0.27-0.81)**
	Adjusted 1	**3.10 (1.71-5.63)**	0.71 (0.16-3.08)	1.06 (0.25-4.59)	**0.25 (0.11-0.59)**	0.64 (0.31-1.35)	0.77 (0.39-1.52)
	Adjusted 2	2.02 (0.97-4.24)	0.61 (0.14-2.64)	1.08 (0.24-4.73)	**0.34 (0.13-0.89)**	0.68 (0.32-1.46)	0.72 (0.34-1.55)
	Adjusted 3	0.68 (0.23-2.06)	0.37 (0.05-3.06)	0.66 (0.07-6.11)	0.47 (0.10-2.25)	0.73 (0.24-2.26)	0.87 (0.31-2.43)
**PFHxA**	Crude	**16.75 (7.09-39.58)**	0.86 (0.01-7.39)	-----	**0.23 (0.07-0.77)**	0.64 (0.35-1.15)	**0.21 (0.11-0.38)**
	Adjusted 1	**12.38 (4.62-33.19)**	1.23 (0.14-11.05)	-----	**0.39 (0.11-1.37)**	0.79 (0.39-1.58)	**0.37 (0.17-0.78)**
	Adjusted 2	**10.53 (3.80-29.23)**	1.29 (0.14-11.56)	-----	0.74 (0.19-2.91)	0.98 (0.47-2.04)	0.63 (0.28-2.04)
	Adjusted 3	**11.01 (3.33-36.45)**	1.51 (0.16-14.67)	-----	1.49 (0.33-6.71)	0.84 (0.31-2.28)	0.63 (0.23-1.73)
**PFPeA**	Crude	**0.41 (0.27-0.63)**	**7.75 (1.79-33.51)**	1.25 (0.46-3.39)	**4.88 (2.14-11.13)**	1.47 (0.87-2.50)	1.37 (0.89-2.10)
	Adjusted 1	**0.34 (0.19-0.60)**	**8.87 (2.01-39.07)**	1.46 (0.51-4.20)	**5.15 (2.19-12.11)**	1.08 (0.60-1.93)	0.74 (0.43-1.29)
	Adjusted 2	**0.30 (0.16-0.56)**	**9.80 (2.20-43.69)**	0.92 (0.30-2.85)	4.06 (1.63-10.13)	0.82 (0.44-1.54)	0.50 (0.27-0.93)
	Adjusted 3	**0.29 (0.15-0.58)**	**12.00 (2.65-54.49)**	1.71 (0.41-7.18)	4.48 (1.67-11.98)	0.96 (0.45-2.02)	0.59 (0.30-1.16)

Adjustment 1	adjusted for year of sample collection, maternal age, parity and timing of sample collection (trimester/cord blood)
Adjustment 2	& race, ethnicity, and education (respectively)
Adjustment 3	& fish consumption and maternal BMI
